# A New Method for Demarcation Line Evaluation: The Corneal Densitometry Graph

**DOI:** 10.3390/jcm15218214

**Published:** 2026-10-22

**Authors:** Yanyan Fu, Jinwen Fu, Yewei Yin, Xi Huang, Aiqun Xiang, Meixia Zhang

**Affiliations:** 1 Department of Ophthalmology and Research Laboratory of Macular Disease, West China Hospital, Sichuan University, Chengdu 610041, China; fyy_666@scu.edu.cn (Y.F.); fujinwen@stu.scu.edu.cn (J.F.); doctorhyh@hotmail.com (X.H.); 2 Eye Center of Xiangya Hospital, Central South University, Changsha 410008, China; 3 Department of Optometry and Ophthalmology, Hunan Provincial People’s Hospital (The First Affiliated Hospital of Hunan Normal University), Changsha 410005, China; xiangaiqun11@163.com

**Keywords:** corneal cross-linking, corneal densitometry, corneal collagen demarcation line, anterior segment optical coherence tomography, in vivo confocal microscopy, agreement

## Abstract

**Objectives:** To evaluate whether the corneal densitometry graph can visualise the demarcation line (DL) and measure its depth early after corneal collagen cross-linking (CXL). **Methods:** A total of 53 eyes of 53 patients with progressive keratoconus underwent accelerated CXL. Visual acuity and corneal topography were measured preoperatively and at 1 month. The apex of the cross-linking reaction (CLR) peak on corneal densitometry graph was compared with the DL depth on anterior segment optical coherence tomography (AS-OCT); the reaction-initiating and reaction-ending depths were compared with those on in vivo confocal microscopy (IVCM). Pearson correlation coefficient was used to assess the linear association between the densitometry-derived depths and the reference measurements. Agreement was evaluated with Bland–Altman analysis together with an absolute-agreement intraclass correlation coefficient (ICC) and changes were analysed with linear mixed-effects models. **Results:** At 1 month postoperatively, uncorrected distance visual acuity (UDVA) did not change significantly (*p* = 0.851), whereas CDVA improved slightly (*p* = 0.022). Corneal densitometry increased in the central region (0–2, 2–6, 6–10 mm) (*p* < 0.001). Corneal densitometry increased significantly in the central region (0–2, 2–6 and 6–10 mm annuli; total layer, all *p* ≤ 0.001) but not in the 10–12 mm annulus (*p* = 0.827). Correlation analysis showed a strong association with AS-OCT and IVCM (r = 0.82–0.88, *p* < 0.001). Agreement with AS-OCT and IVCM was good at the group level (ICC(2,1), 0.82–0.87), although all 95% limits of agreement exceeded the ±5% limit. A CLR peak was identified in all 53/53 eyes versus 50/53 eyes on AS-OCT, but the detection rates did not differ significantly (McNemar exact test, *p* = 0.25). **Conclusions:** The corneal densitometry graph provides an initial, non-contact evaluation of the depth and extent of the early cross-linking reaction, with good group-level agreement with AS-OCT and IVCM.

## 1. Introduction

Corneal collagen cross-linking (CXL) is a noninvasive therapeutic approach to corneal diseases [1]. By applying the riboflavin solution to the corneal surface followed by ultraviolet-A (UVA) irradiation, cross-links are formed between the collagen fibrils of the corneal stroma [2,3]. The procedure stiffens the anterior corneal stroma, thereby improving corneal biomechanics and halting ectatic progression [4], and it is now regarded as a standard, minimally invasive and safe treatment for progressive keratoconus [5,6]. Long-term studies have reported improvements in refractive and topographic outcomes. However, because of early cross-linking reactions, many corneal morphological parameters (e.g., K value and corneal pachymetry) worsened in the early period [7,8]. Refractive surgeons rely mainly on these corneal-topography parameters to monitor corneal morphology and to estimate the postoperative prognosis [9]. Early assessment of the cross-linking reaction is therefore of considerable importance to both surgeons and patients. The corneal stromal demarcation line (DL) is the most readily observable corneal response in this early period.

The DL is a hyper-reflective line within the corneal stroma that can be detected on slit-lamp examination as early as 2 weeks after CXL and is considered to mark the boundary between the cross-linked and untreated regions [10]. Since Cannon et al. first described this line in 1978 [11,12], researchers have subsequently used anterior segment optical coherence tomography (AS-OCT) to visualise and measure the depth of the DL [13,14]. They found that the DL depth following epi-off CXL (≈300–350 µm) [15] was deeper than that following transepithelial epi-on CXL (≈150–210 µm) [16]. On the basis of the CXL mechanism and the Lambert–Beer law [17], some authors have proposed that “the deeper, the better”, although whether a deeper reaction predicts better postoperative efficacy remains debated. To characterise the reaction at the cellular level, in vivo confocal microscopy (IVCM) has been used to visualise ultrastructural changes in the treated stroma [12]. IVCM shows that the DL corresponds to a “cross-linking reaction zone” formed by the aggregation of apoptotic keratocytes, lacunar corneal oedema and activated keratocyte nuclei [18], which renders this region highly reflective [19]. Because IVCM requires direct corneal contact, however, many clinicians are reluctant to use it in the early postoperative period [20]. AS-OCT is more widely used clinically because it avoids the risk of infection [14], but we have encountered eyes with a typical cross-linking reaction on IVCM in which no clear DL could be identified on AS-OCT. The practical evaluation of efficacy and safety after CXL therefore remains exploratory, and surgeons would benefit from a simple, intuitive means of assessment in the early postoperative period.

Corneal densitometry is a measurement module of the Scheimpflug topography system and is inversely related to corneal transparency [21,22]. It has previously been used to assess subepithelial haze and stromal scarring after refractive surgery and keratoplasty [23], and it is increasingly applied to the evaluation of CXL outcomes [24]. In addition to showing the corneal densitometry of each layer, it also provides the average and maximal densitometry graphs as a function of cornea depth from the epithelium to the endothelium. This DL phenomenon appears differently on each of the three different instruments: the demarcation line (DL) appears on AS-OCT as a hyper-reflective stromal interface; the cross-linking reaction zone is visualised by IVCM as a band of increased reflectivity with a start and an end; and the cross-linking reaction peak (CLR peak) proposed here appears as a new local on the maximum-densitometry curve of the Scheimpflug system. To our knowledge, this derived graph has received little attention. After a systematic comparison of corneal densitometry data, we observed that the maximum corneal densitometry graph (the corneal densitometry graph) offers a new and informative way to visualise the extent and depth of the cross-linking reaction. We therefore designed this study to evaluate this method as an initial approach to assessing the depth of the cross-linking reaction in the early period after CXL and to compare it with AS-OCT and IVCM.

## 2. Methods

### 2.1. Patients

This retrospective clinical study included 53 eyes of 53 patients with progressive keratoconus who underwent accelerated CXL. Of these, 16 patients had bilateral disease meeting the inclusion criteria. To avoid confounding from inter-eye correlation, only one eye per patient was included in the analysis, and, for patients with bilateral involvement, the eye was selected at random. All the patients were diagnosed on the basis of corneal topography (Pentacam, Oculus, Wetzlar, Germany) and slit-lamp examination, and underwent Avedro accelerated CXL at the Department of Ophthalmology, Xiangya Hospital, Central South University, Changsha, Hunan, China, between 2023 and February 2026. The included eyes underwent Avedro accelerated CXL from June 2023 to February 2026. The study was approved by the Institutional Review Board and conducted in accordance with the tenets of the Declaration of Helsinki. All patients gave their informed consent before participating.

The inclusion criteria were as follows: (I) aged between 10 and 40 years; (II) progressive keratoconus, diagnosed on the basis of one or more of the following changes during the preceding 6 to 12 months: an increase of 1.00 dioptre (D) or more in the steepest keratometry (Kmax) reading on corneal tomography, an increase of 1.00 D or more in the manifest cylinder, or an increase of 0.50 D or more in the manifest refraction spherical equivalent; and (III) a corneal thickness greater than 400 μm. Included eyes were classified as stages I to III according to the Amsler–Krumeich classification [25] The exclusion criteria were as follows: (I) a corneal thickness of less than 400 μm; (II) a K-avg value greater than 60 D; and (III) a history of ocular surgery or of ocular pathological signs, such as herpetic keratitis, dry eye, corneal infection, ocular or systemic autoimmune disease, pregnancy or lactation.

### 2.2. Ethical Statement

The study was conducted in accordance with the Declaration of Helsinki, and approved by the Ethics Committee of Xiangya Hospital, Central South University (approval number 201612074, date of approval 30 December 2016). Informed consent was obtained from all subjects involved in the study.

### 2.3. Surgical Technique

All procedures were performed by the same surgeon. The epi-off CXL technique was performed after topical anaesthesia with 0.4% oxybuprocaine hydrochloride eye drops (Santen Pharmaceutical Co., Ltd., Osaka, Japan); and the epithelium was removed with a blunt metal spatula over an 8- or 9-mm circle after the cornea had been soaked in 20% alcohol for 20 s. Riboflavin 0.1% (VibeX Rapid, Avedro Inc., Waltham, MA, USA) was applied topically every 2 min for 30 min and was continued every 2 min during UVA exposure. The cornea was exposed to 370-nm UVA light (Avedro System, Avedro Inc., Waltham, MA, USA) for 10 min at an intended irradiance of 9 mW/cm^2^ (5.4 J/cm^2^ surface dose) (Supplementary Table S1). At the end of the procedure, a soft therapeutic contact lens was applied until complete re-epithelialisation.

### 2.4. Corneal Topography and Corneal Densitometry

Corneal topography and densitometry data were obtained with a Scheimpflug topography system (Pentacam HR, Oculus Optikgeräte GmbH, Wetzlar, Germany) preoperatively and 1 month postoperatively. Corneal densitometry measures the central corneal area in a 12-mm diameter zone and expresses it in grayscale units ranging from 0 (optically clear cornea) to 100 (total corneal opacity). This zone is divided by concentric circles into four annuli (0–2, 2–6, 6–10 and 10–12 mm) and, by corneal depth, into an anterior layer (up to 120 µm), a posterior layer (the posterior 60 µm) and a central layer (between the anterior and posterior layers).

**Figure 1:**
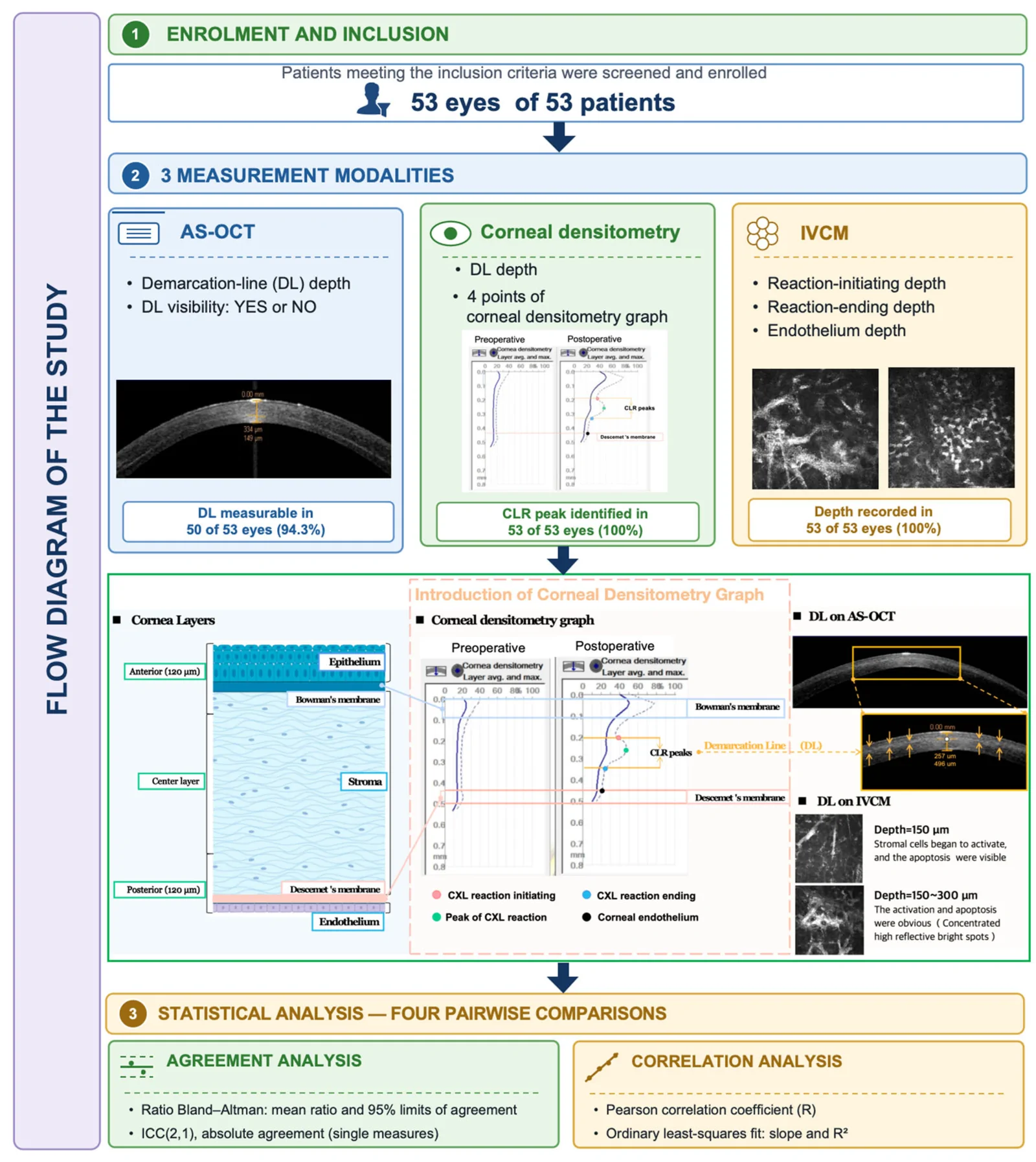
Flow diagram of the study. Flow diagram of the 53 included eyes and an introduction to the corneal densitometry graph: left: diagram of the corneal layers; centre: the corneal densitometry graph; right: DL on AS-OCT and IVCM. The blue dotted line represents the maximum corneal densitometry value, and the solid line represents the average corneal densitometry value. Bowman’s and Descemet‘s membranes on the graph; the fibres in Bowman’s and Descemet ‘s membrane are arranged closely, so the densitometry of these two layers is higher than that of the intermediate corneal stroma. Two peaks are present on the blue line both pre- and postoperatively, approximately at the depth of the Bowman’s membrane (0–50 um) and Descemet ‘s membrane (450–500 um). In addition to these two peaks, the new peak(s) appeared on the blue dashed curve at 1 month after CXL; we named this phenomenon the cross-linking reaction peaks (CLR peaks). Red dot: CXL reaction-initiating point; the vertical coordinates of the red dots as the starting depth of the cross-linking reaction measured by the corneal densitometry graph; green dot: vertex point of the cross-linking reaction peak(s); the vertical coordinates of the green dots is the DL depth measured by the corneal densitometry graph; blue dot: CXL reaction-ending point; the vertical coordinate of the blue dots as the stopping depth of cross-linking reaction measured by the corneal densitometry graph; black dot: starting point of the corneal endothelium, depth between the blue and black dot vertical coordinates is the distance between the cross-linking reaction and the corneal endothelium.

The corneal densitometry graph provided by the Scheimpflug topography system is a function of corneal depth: the vertical coordinate represents corneal depth (in µm), and the horizontal coordinate represents the corneal densitometry value (in percentage grayscale units). The graph contains two curves: the blue dotted line represents the maximum corneal densitometry value and the solid blue line represents the average corneal densitometry value (Figure 1). Because the fibres of Bowman’s membrane and Descemet’s membrane are closely packed, the densitometry of these two layers is greater than that of the intermediate corneal stroma [26]. Consequently, both pre- and postoperative densitometry graphs show two peaks, located approximately at the depth of Bowman’s membrane (≈50 µm from the anterior surface of the epithelium) and of Descemet’s membrane (≈450–500 µm in normal eyes; in keratoconic eyes ≈ 50 µm from the posterior surface of the endothelium). After CXL, new peak(s) appeared on the blue dotted line in addition to these two peaks; we refer to them collectively as the cross-linking reaction peak group (CLR peaks), while acknowledging that backscatter changes after CXL may also reflect oedema keratocyte activation and stromal remodelling (Figure 1). The presence of CLR peaks indicates a visible positive result for the cross-linking reaction. When the blue dotted lines obtained before and after the operation were compared, the appearance of visible CLR peak(s) was recorded as “yes” and its absence as “no” (Supplementary Table S2). The coordinate reading of these peaks is described in the next section.

### 2.5. Coordinate Reading, Calibration and Repeatability

Densitometry images were exported from the Pentacam HR system at a fixed pixel resolution (Supplementary Figure S1A). We used dedicated coordinate software to convert the graph coordinates into absolute depth values for the DL and for the cross-linking reaction zone. The full corneal thickness measured by corneal topography was used to calibrate the absolute difference between the start and end points of the corneal densitometry graph. The aspect ratio of each rotated image was verified before measurement (Supplementary Figure S1B,C).

All readings were performed independently by two trained operators (Yanyan Fu and Aiqun Xiang), and disagreements were resolved by consensus; inter-observer agreement for peak identification was assessed by κ statistic. A CLR peak was defined as a new local maximum on the postoperative maximum corneal densitometry curve that satisfied all of the following criteria: (i) no corresponding peak was present at the same depth on the preoperative curve; (ii) the densitometry value at that depth exceeded the preoperative value by at least 5 percentage units (≥5%); and (iii) the peak spanned a depth interval of at least 20 µm between the two points at which the curve crossed the preoperative reference value (defining the boundaries of the CLR peak). When more than one candidate peak was present (Supplementary Figure S1D), the peak with the largest amplitude increase relative to the preoperative curve was selected for depth analysis, whereas all detected peaks were recorded for the visibility assessment. Peaks that could not be reliably distinguished from the pre-existing Bowman or Descemet peaks were classified as ambiguous and excluded from depth analysis.

Once the CLR peak was confirmed, we manually selected the following landmarks on the curve: the CLR-peak vertex (green dot in Figure 1); the reaction-initiating point (red dot); the reaction-ending point (blue dot); and the Descemet’s membrane endpoint (black dot). The vertical coordinate of the CLR-peak vertex (green dot) represented the depth of the cross-linking DL; the vertical coordinates of the reaction-starting (red dot) and reaction-ending (blue dot) points represented the initial and terminal depths of the cross-linking reaction; and the vertical coordinate of Descemet’s membrane (black dot) represented the starting depth of the corneal endothelial layer. Measurement repeatability was assessed by re-reading a subset of images. The coordinate software is available from the corresponding author upon reasonable request.

### 2.6. AS-OCT

The corneal stromal DL was observed on the AS-OCT images (Tomey, CASIA, Japan) using the anterior segment scan protocol. Images were centred on the corneal vertex, and images with motion artefact or an incomplete stromal outline were excluded. Two independent observers (Jinwen Fu and Yewei Yin) recorded its visibility and measured its depth with the flap tool. The mean DL depth was obtained by averaging the recorded depth at 90° and 180° for a reproducible reference and to avoid the variable cone apex in keratoconus [13], and this value was expressed as a percentage of the total corneal thickness measured at the same location (Supplementary Figure S2).

### 2.7. IVCM

**Figure 2:**
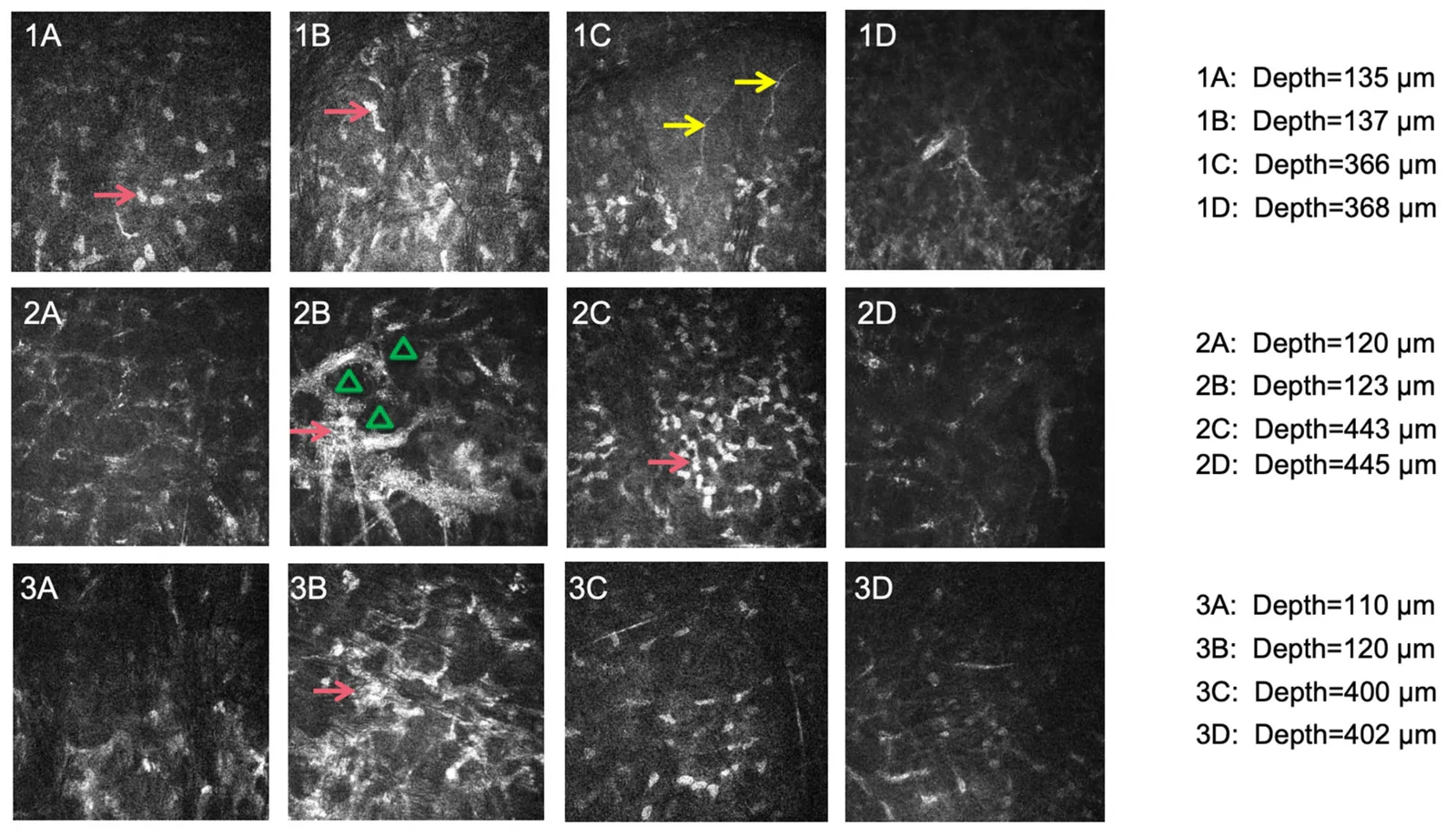
Depth of the cross-linking reaction measured by IVCM. (**1A**–**3D**): postoperative ultrastructural pictures of the cornea at various depths (the depth value corresponding to each image is marked on the right side). Red arrow: activated keratocyte nuclei (multiple activated cells form a brightness unit). Yellow arrow: nerve fibres; green triangle: lacunar corneal edemaA and B are two pictures of adjacent depths on the IVCM. B has five or more brightness units than A. The depth of A serves as the starting point of the cross-linking reaction. C and D are two pictures of adjacent depths on the IVCM. D has no brightness units compared with C. The depth of D serves as the ending point of the cross-linking reaction.

IVCM images (ConfoScan 4, Nidek Technologies S.r.l., Padua, Italy) were acquired over the central cornea (a 2 mm diameter area at the cornea apex), from the anterior surface of the epithelium to the posterior surface of the endothelium. Two independent observers (Jinwen Fu and Yewei Yin) recorded the depths of the corneal stroma at which the cross-linking reaction started and ended and calculated these depths as a percentage of the total corneal thickness. The depth of the cross-linking reaction was measured as described previously [12]: as the highly reflective bright area on IVCM increased, the depth at which the cross-linking reaction started was defined as the depth at which an increase of 5 brightness units or more occurred between two sequential images, and the depth of the first of these images was taken as the starting depth. Similarly, when no highly reflective bright area was observed in two consecutive IVCM images, the depth of the last image was taken as the reaction-ending depth (Figure 2). Both observers were masked to the findings and to each other’s measurements.

### 2.8. Outcomes and Statistical Analysis

Data and outcomes. Data obtained from patient records preoperatively and 1 month after CXL included age, sex, uncorrected visual acuity (UCVA) and corrected distance visual acuity (CDVA), together with corneal topography parameters (keratometry, thickness of the apex point and thinnest points, elevation of the apex and thinnest points, and corneal densitometry). One month after CXL, the depth of the cross-linking reaction was measured with three instruments: AS-OCT (depth of the DL); IVCM (depth of the reaction-initiating and reaction-ending points of the cross-linking reaction, and depth of the endothelium); and corneal densitometry graph (vertical coordinates of the reaction-initiating and reaction-ending points and of the Descemet’s membrane). Inter-observer reliability for the depth measurements was quantified with the intraclass correlation coefficient ICC(2,1).

Correlation and agreement. The Pearson correlation coefficient (R) was used to assess the strength and direction of the linear association between corneal densitometry and the reference measurements (AS-OCT and IVCM). Agreement was assessed using ratio Bland–Altman analysis [13,27] (the ratio of the two methods [28], densitometry divided by the reference, plotted against their mean; 95% limits of agreement on the ratio scale) and with the two-way random-effects, absolute-agreement intraclass correlation coefficient ICC (2,1) [29].

Statistical models and multiplicity. Because the inter-method difference was proportional rather than a fixed offset, all depths were expressed as a percentage of the corresponding full corneal thickness, and a pre-specified clinically acceptable limit of ±5% relative difference (ratio 0.95–1.05) was used to judge interchangeability. Because the analysis included one eye per patient, each eye was treated as an independent unit of analysis and no adjustment for fellow-eye correlation was required. For continuous outcomes, linear mixed-effects models with a random intercept for patient were fitted; the patient random intercept accommodates the repeated pre- and postoperative measurements within the same eye. For the binary detection outcome, the exact McNemar test was used at the eye level as a within-eye paired comparison of AS-OCT and corneal densitometry. Eyes without a measurable value on a given modality were excluded from that pairwise comparison. Because of the large number of comparisons in the densitometry analysis, *p*-values were adjusted with the Benjamini–Hochberg procedure (FDR = 0.05). Expressing depth as a percentage of corneal thickness also removes between-device differences in absolute depth scaling, so that the compared values refer to the same relative depth rather than to an absolute micrometre level. Analyses were performed with Python (version 3.x, Python Software Foundation, Wilmington, DE, USA; SciPy and statsmodels libraries) and IBM SPSS Statistics version 27.0 (IBM Corp., Armonk, NY, USA); 95% CIs for the ICC were obtained by non-parametric bootstrapping (2000 resamples). *p*-values of less than 0.05 were considered statistically significant.

## 3. Results

### 3.1. Demographic Characteristics of the Included Eyes

A total of 53 eyes from 53 patients met the inclusion criteria and had complete imaging at the 1-month visit, with only one eye per patient included. The demographic characteristics and preoperative parameters are presented in Table 1.

### 3.2. Visual Acuity

One month after CXL, UDVA showed no significant change from baseline (*p* = 0.851), whereas CDVA improved slightly yet significantly (*p* = 0.022) (Table 2).

### 3.3. Changes in Corneal Morphology

At the 1-month follow-up, Kmax had increased significantly (*p* = 0.001). The anterior-surface K-flat, K-steep and K-average values also increased significantly (all *p* < 0.001), whereas anterior-surface astigmatism (*p* = 0.639) and all posterior-surface keratometry values (K-flat, *p* = 0.121; K-steep, *p* = 0.716; K-average, *p* = 0.452; astigmatism, *p* = 0.682) showed no significant change. The thickness at the apex point and at the thinnest point had decreased significantly (−13.08 µm; both *p* < 0.001). The anterior elevation of the apex point increased significantly (*p* < 0.001), whereas the posterior elevation of the apex point (*p* = 0.069) and the anterior and posterior elevations of the thinnest point (*p* = 0.692 and *p* = 0.720, respectively) showed no significant change (Table 2).

### 3.4. Changes in Corneal Densitometry

One month after accelerated CXL, corneal densitometry had increased significantly in all layers of the 0–2 mm and 2–6 mm annuli (all *p* < 0.001) and in most layers of the 6–10 mm annulus (anterior layer, *p* < 0.001; middle layer, *p* = 0.003; total, *p* = 0.001), whereas the posterior layer of the 6–10 mm annulus (*p* = 0.402) and all layers of the 10–12 mm annulus (*p* = 0.334–0.827) showed no significant change (Table 3 and Supplementary Figure S3).

### 3.5. Depth of Demarcation Line and Cross-Linking Reaction Zone

The visualisation (YES or NO) and depth of the DL measured by AS-OCT and the corneal densitometry graph are presented in Supplementary Table S2. Of the 53 eyes, AS-OCT measured the DL in 50 eyes (94.3%; 95% CI, 84.6–98.1%), whereas a cross-linking reaction peak appeared on the corneal densitometry graph in all 53 eyes (100%; 95% CI, 93.2–100%). The detection rates did not differ significantly (three discordant pairs; McNemar exact test, *p* = 0.25). In the three eyes in which AS-OCT showed no visible DL, the CLR peak identified by densitometry was located at 189.7 µm (range, 48–298 µm), corresponding to 41.7% of the corneal thickness. Because no independent reference standard exists for the presence of a cross-linking reaction, we report these proportions are reported as detection rates rather than as diagnostic sensitivities (Supplementary Table S3 and Table 4).

**Figure 3:**
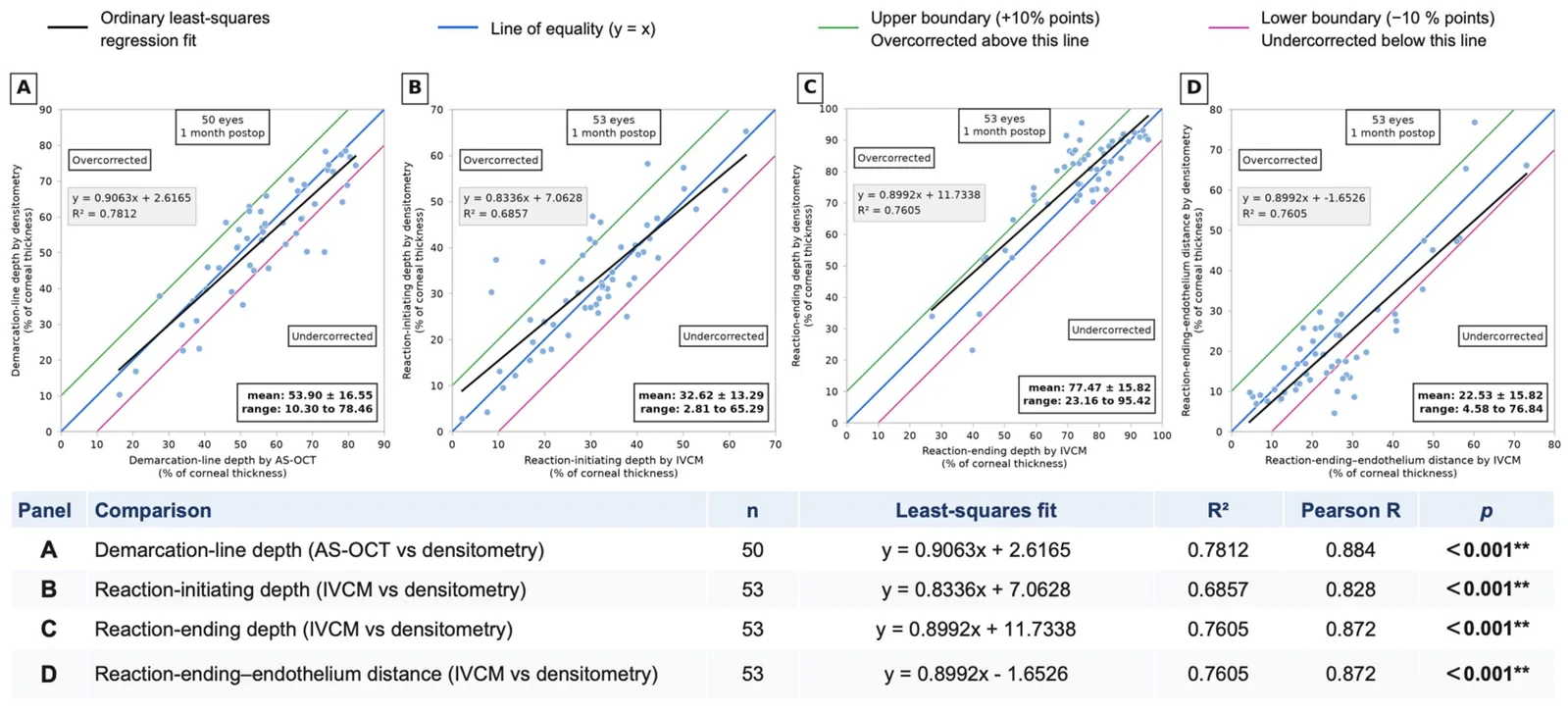
Correlation analysis of the depth value measured by the corneal densitometry graph versus AS-OCT/IVCM. (**A**): Scatter plots with the depth of the DL measured by AS-OCT on the X-axis and the depth of the DL measured by the Max corneal densitometry graph on the Y-axis. (**B**): Scatter plots with the depth of reaction initiation measured by IVCM on the X-axis and the depth of reaction initiation measured by the Max corneal densitometry graph on the Y-axis. (**C**): Scatter plots with the depth of reaction ending measured by IVCM on the X-axis and the depth of reaction ending measured by the Max corneal densitometry graph on the Y-axis. (**D**): Scatter plots with the distance from the reaction-ending point to the corneal endothelium measured by IVCM on the X-axis and the same distance measured by corneal densitometry on the Y-axis. In each panel, the black line represents the ordinary least-squares regression fit, with the regression equation and the coefficient of determination (R^2^) shown in the inset; the blue line is the line of equality (y = x); the green line marks the upper boundary, 10 percentage points of corneal thickness above the line of equality, beyond which the densitometry-derived depth exceeds the reference measurement by more than 10 percentage points (“Overcorrected”); the magenta line marks the lower boundary, 10 percentage points below the line of equality, below which the densitometry-derived depth is more than 10 percentage points smaller than the reference measurement (“Undercorrected”); data points lying between the green and magenta lines are within ±10 percentage points of corneal thickness of the reference measurement. The black boxes report the mean ± standard deviation and the range of the depth values measured by the corneal densitometry graph in each panel. The number of eyes is shown in each panel *n* = 50 in (**A**), as the DL was visible on AS-OCT in 50 of 53 eyes; *n* = 53 in (**B**–**D**). **: *p* < 0.001.

The Pearson correlation coefficients for the four comparisons were 0.884, 0.828, 0.872 and 0.872 (all *p* < 0.001); the corresponding scatter plots are provided for reference (Figure 3A–D), but we report correlation only as a secondary descriptor (Table 4). Agreement between the methods was assessed using ratio Bland–Altman analysis and the absolute-agreement intraclass correlation coefficient ICC (2,1). Because the inter-method difference was proportional rather than a fixed offset, the Bland–Altman ordinate was the ratio rather than the difference, and all depths were expressed as a percentage of the corresponding full corneal thickness. A pre-specified clinically acceptable limit of ±5% relative difference (ratio 0.95–1.05) was used to judge whether the two methods could be used interchangeably.

**Figure 4:**
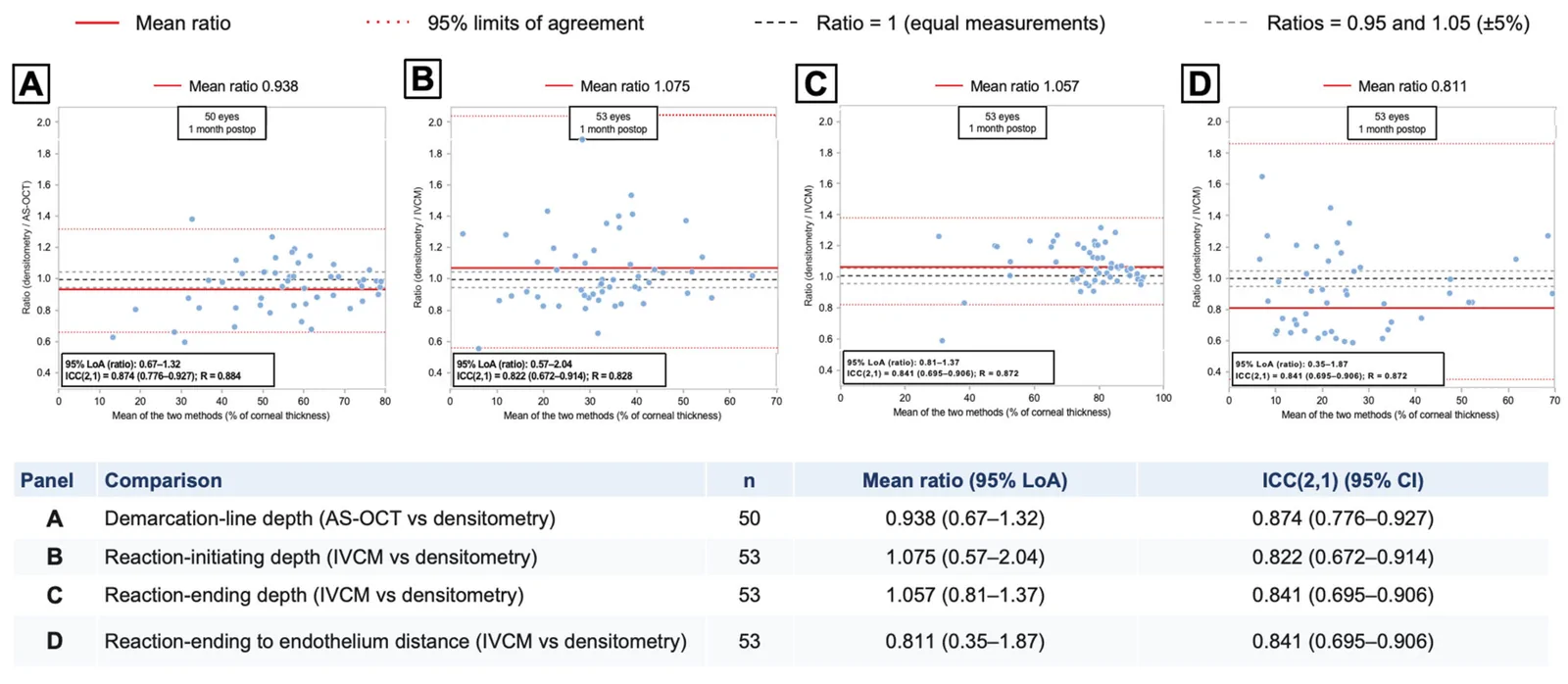
Agreement analysis of the depth value measured by the corneal densitometry graph versus AS-OCT/IVCM. Each panel plots the ratio of corneal densitometry to the reference method (Y-axis) against the mean of the two percentages of corneal thickness (X-axis). (**A**): Ratio Bland-Altman plot for the DL depth measured by the densitometry graph versus AS-OCT (*n* = 50). (**B**): Ratio Bland-Altman plot for the reaction-initiating depth measured by the densitometry graph versus IVCM (*n* = 53). (**C**): Ratio Bland-Altman plot for the reaction-ending depth measured by the densitometry graph versus IVCM (*n* = 53). (**D**): Ratio Bland-Altman plot for the distance from the reaction-ending point to the corneal endothelium measured by the densitometry graph versus IVCM (*n* = 53). In each panel, the solid red line denotes the mean ratio (corneal densitometry ÷ reference method); the red dotted lines denote the 95% limits of agreement (LoA) on the ratio scale; the grey dashed lines at ratio 0.95 and 1.05 denote the pre-specified ±5% acceptable relative-difference band; the black dashed line at ratio 1.0 denotes the line of equality (perfect agreement). ICC(2,1) = two-way random-effects, absolute-agreement intraclass correlation, 95% CIs by non-parametric bootstrap. The table summarises the comparison data for each group.

The results are summarised in Table 4 and Figure 4:

(I) For DL depth, the 50 eyes with a measurable value on both modalities showed 56.6% ± 16.1% (AS-OCT) and 53.9% ± 16.6% (corneal densitometry) of the corneal thickness. The mean ratio (densitometry divided by AS-OCT) was 0.938 (95% limits of agreement on the ratio scale, 0.67–1.32), and the absolute-agreement ICC (2,1) was 0.874 (95% CI, 0.776–0.927).
(II) For the depth at which the cross-linking reaction was initiated, IVCM gave 30.7% ± 13.2% and corneal densitometry 32.6% ± 13.3% of the corneal thickness in all 53 eyes; the mean ratio (densitometry divided by IVCM) was 1.075 (95% LoA ratio, 0.57–2.04) and the ICC (2,1) was 0.822 (95% CI, 0.664–0.912).
(III) For the depth at which the reaction ended, IVCM gave 73.1% ± 15.3% and corneal densitometry 77.5% ± 15.8%; the mean ratio was 1.057 (95% LoA ratio, 0.81–1.37) and the ICC (2,1) was 0.841 (95% CI, 0.702–0.903).
(IV) For the distance from the termination of the reaction to the corneal endothelium, IVCM gave 26.9% ± 15.3% and corneal densitometry 22.5% ± 15.8% of the corneal thickness. The mean ratio (densitometry divided by IVCM) was 0.811 (95% LoA ratio, 0.35–1.87), and the ICC (2,1) was 0.841 (95% CI, 0.701–0.906).

Taken together, the mean ratios were close to 1.00 for the DL depth (0.94), the reaction-initiating depth (1.08), and the reaction-ending depth (1.06), whereas the ending-point-to-endothelium ratio was 0.81; the ICC(2,1) values indicated good agreement at the group level (ICC, 0.82–0.87). However, the 95% limits of agreement on the ratio scale were wide and exceeded the pre-specified acceptable band of 0.95–1.05 in all four comparisons (ratio LoA ranging from 0.35 to 2.04). Therefore, the two methods provide concordant information at the group level but cannot be regarded as interchangeable for the assessment of an individual eyes; corneal densitometry should be regarded as a useful complement to, rather than a substitute for, AS-OCT and IVCM.

### 3.6. Observer Agreement and Repeatability

Inter-observer agreement for the identification of a cross-linking reaction (CLR) peak was assessed with the κ statistic and was κ = 0.85 (95% CI, 0.74 to 0.93) across 53 eyes. Measurement repeatability was assessed by re-reading all the images after 2 weeks; the within-subject standard deviation of the differences was 8.5 µm and the repeatability coefficient (1.96 × SD) was 16.7 µm. The intraclass correlation coefficient ICC(2,1) for the demarcation-line depth was 0.92 (95% CI, 0.88 to 0.95) for AS-OCT and 0.88 (95% CI, 0.82 to 0.93) for corneal densitometry. The inter-observer reliability for the IVCM depth readings were as follow: ICC(2,1) = 0.571 (95% CI, 0.174 to 0.988; *n* = 53 eyes), with a mean inter-observer difference of −10.80 µm (SD of differences 43.21 µm) and 95% limits of agreement −95.49 to +73.89 µm. Because each patient contributed only one eye, there was no fellow-eye clustering in the primary analysis, and the group-level agreement reported here is not an artefact of including fellow eyes.

## 4. Discussion

The safety and efficacy of CXL remain central concerns for refractive surgeons [30], yet a visible corneal improvement after treatment appears slowly [30,31]. Early postoperative monitoring and prediction of the cross-linking reaction are therefore important [32,33]. This study focused on the early biological and morphological changes and on the depth of the cross-linking reaction after CXL, aiming to identify better indicators or methods for postoperative evaluation of efficacy and safety.

In this study, anterior-surface keratometry values increased 1 month after CXL, consistent with other short-term reports [34,35]. Long-term studies [36,37] have shown that keratometry values initially increase after CXL and then decline, returning to preoperative levels by 3–6 months before continuing to decrease; approximately 12 months are required before a significant difference from preoperative values becomes apparent. The initial increase in keratometry is probably more likely to reflect the early stromal oedema, hydration changes, and remodelling that follow epithelial debridement and UVA/riboflavin exposure, which transiently alter the anterior corneal surface and its measured curvature [38]. Subsequently, the covalent bonds formed during cross-linking tighten the collagen fibrils, compress the corneal stroma, and flatten the cone, and keratometry decreases only at 6–12 months postoperatively. The changes at the apex and thinnest points followed the same pattern: at 1 month, the thickness at both locations had decreased significantly and the anterior elevation of the apex had increased, indicating a forward shift of the anterior corneal surface. At early postoperative review, these morphological parameters therefore tend to appear worse. Long-term studies indicate that this early trend does not represent a genuinely worse outcome and may simply reflect normal epithelial remodelling [13,39]. Early corneal morphological changes alone are therefore insufficient to predict CXL efficacy.

To estimate the efficacy and safety of epi-off CXL at an early stage, surgeons have focused on the depth of the DL as an indicator of the postoperative response. AS-OCT [40] and IVCM [12] are currently the methods most commonly used for this measurement, but both have practical limitations. The appearance of the DL on AS-OCT is variable: a clearly hyper-reflective line is usually visible 2 weeks to 1 month after CXL, but in some eyes no clear DL can be identified [41], or only an indistinct band is seen within the corneal stroma [12]. Ozgurhan et al. [42] reported that eyes without a clear DL on AS-OCT still showed typical cross-linking reactions on IVCM (apoptotic keratocytes, lacunar corneal oedema and activated keratocyte nuclei). The detection rate of AS-OCT for the cross-linking reaction is therefore not complete. Microscopically, the “demarcation line” corresponds to a cross-linking reaction zone that can be visualised with IVCM [10]; however, IVCM requires direct corneal contact and is not generally recommended in the early postoperative period because of the associated risk of infection [43]. In our study, the corneal densitometry graph identified a cross-linking reaction peak in a higher proportion of eyes than AS-OCT (100% vs. 94.3%), although this difference was not statistically significant (*p* = 0.25).

Corneal densitometry has gradually attracted researchers’ attention. Several groups [44,45,46] have reported increased corneal densitometry after CXL, and our results show the same trend. Several mechanisms have been proposed to explain the loss of stromal transparency after CXL: increased collagen fibril diameter [47], the cellular reaction induced by keratocyte activation [20] and changes in the refractive index of the stroma [12,48]. Some authors have suggested that the magnitude of the densitometry change is proportional to the extent of the cross-linking reaction. Densitometry alone, however, provides only a preliminary description of changes in corneal transparency and cannot by itself quantify the extent of the cross-linking reaction.

Because cross-linking forms new chemical bonds between collagen fibrils, increasing corneal densitometry and reducing transparency, we hypothesised that this change in the Max corneal densitometry graph reflects the cross-linking reaction and may therefore inform postoperative evaluation. To examine this, we used coordinate software to convert the graph coordinates into depth values for the DL and compared these depths with those from AS-OCT and IVCM in two complementary ways. Correlation analysis (Figure 3) quantified the strength and direction of the linear association between measurements, and agreement analysis (Figure 4) assessed whether the methods could be used interchangeably, because a high correlation coefficient describes only association: neither a high r nor the absence of a significant difference between means indicates equivalence.

In the correlation analysis, the depths derived from the Max corneal densitometry graph were strongly and linearly associated with the reference measurements across the full depth range of the cross-linking reaction (Pearson r = 0.83–0.88; all *p* < 0.001) (Figure 3). The regression slopes (0.83–0.91) and coefficients of determination (R^2^ = 0.69–0.78) indicated that the densitometry graph captured most of the variance in the reference measurements: for the DL depth, the graph slightly underestimated deeper values (slope, 0.91), whereas, for the reaction-initiating and reaction-ending depths, the relationship was proportional (slopes, 0.83 and 0.90) with positive intercepts. This proportionality of the inter-method relationship shows that the two instruments scale depth in a systematically similar but not identical manner—precisely the situation in which a correlation coefficient alone would overstate agreement and a ratio-based agreement analysis adds necessary information. The agreement analysis showed mean ratios close to 1.0 for DL depth (0.94), reaction-initiating depth (1.08), and reaction-ending depth (1.06), and the ICC(2,1) values (0.82–0.87) indicated good group-level agreement (Figure 4). However, the 95% limits of agreement on the ratio scale exceeded the pre-specified ±5% limit in all four comparisons (ratio LoA, 0.35–2.04). The densitometry graph and reference modalities therefore agree well at the group level but are not interchangeable for individual eyes.

Furthermore, when the DL was not clearly visible on AS-OCT 1 month after CXL, the CLR peak remained present on the corneal densitometry graph in the same eye at the same visit (3 of 53 eyes; Supplementary Table S3). Theoretically, AS-OCT images are formed by dynamic light scattering, and tissues of differing density at different depths produce different scattering patterns. When the cross-linking reaction is too subtle to distinguish from adjacent tissue, the DL may be difficult to identify on AS-OCT, whereas the same subtle reaction may be more apparent as a change in densitometry—particularly as a change in the slope of the Max corneal densitometry graph. Because small densitometric changes are amplified when plotted as a curve and read with coordinate software, the reaction was detected in a higher proportion of eyes. Whether this translates into superior clinical performance requires further study.

In addition, the concept of the “cross-linking reaction zone” was emphasised in this study. We selected the starting and ending points of this zone from IVCM and compared them with the corresponding points of the CLR peak(s) on the corneal densitometry graph. The two methods showed good group-level agreement, although the limits of agreement were wide. The advantage of IVCM is that it expands the “demarcation line” into a “cross-linking reaction zone,” and the corneal densitometry graph can depict the same zone through the information contained in the curve. Moreover, the new method is non-contact and therefore avoids the infection risk associated with contact measurements.

Postoperative safety is another important consideration. Some studies have proposed that the “deeper, the better” principle can be guide postoperative evaluation of CXL [13,47], whereas others have questioned this view because of the risk of damage to adjacent tissue. Endothelial cell damage has been reported after epi-off CXL [49], and lower endothelial cell density may occur when the treatment depth is excessive; this is why we required a minimum corneal thickness for inclusion. The “deeper, the better” principle can therefore be applied to efficacy only within the limits of safety. Using the coordinate software, we found that the corneal densitometry graph can also be used to measure the distance from the cross-linking reaction to the corneal endothelium, with group-level agreement similar to that obtained with IVCM. These findings suggest that the method may provide useful information for assessing postoperative safety, although the present data do not permit a direct comparison of clinical safety between measurements.

For clinical use, the coordinate software is not essential: surgeons can obtain at least three pieces of information by inspecting the corneal densitometry graph directly. First, the presence of CLR peaks indicates that a cross-linking reaction has occurred. Second, the approximate depth of the cross-linking reaction zone can be estimated from the vertical range of the peaks. Third, the distance from the reaction zone to the corneal endothelium can be estimated. Some recent studies have suggested that the horizontal coordinate of the CLR peak(s) may indirectly reflect the extent of the cross-linking reaction. The method is simple and non-contact and avoids the direct corneal contact required by IVCM; we did not assess comparative clinical safety in this study. The same instrument also provides essential biometric measurements, such as corneal curvature and thickness.

Limitations. This study should be regarded as an initial evaluation of the proposed method. The densitometric features were both identified and assessed in the same cohort, and no independent validation sample was available; the generalisability of the findings therefore requires confirmation in an external cohort. Because no suitable control group was available, the specificity of the corneal densitometry graph for the cross-linking reaction cannot be established, and the peak may in part reflect non-specific postoperative stromal changes such as oedema and remodelling. In addition, this was a single-centre study using one accelerated epi-off protocol, and all measurements were obtained at a single postoperative time point. Whether the corneal densitometry graph predicts the long-term efficacy of CXL cannot be determined from the present data. Finally, the three imaging examinations were performed at a single postoperative visit, so test–retest variability over time could not be assessed.

## 5. Conclusions

In summary, the corneal densitometry graph may complement, rather than replace, AS-OCT and IVCM in the early assessment of the cross-linking reaction. It translates the concept of the “cross-linking demarcation line” into a “cross-linking reaction zone” and, from a single convenient and non-contact examination, provides information on early morphological changes, the depth of the cross-linking reaction, and its distance from the corneal endothelium. Depths derived from the corneal densitometry graph were strongly correlated with the AS-OCT and IVCM measurements and showed good group-level agreement. The method should therefore be regarded as an initial evaluation; further evidence from external cohorts and long-term studies is required to confirm its accuracy and reproducibility.

## Figures and Tables

**Table 1:** Demographic Characteristics and Preoperative Parameters of the Included Eyes (*n* = 53).

Characteristic	Value
Sex (M/F)	31/22
OD/OS	37/16
Age, years (min, max values)	20.02 ± 5.03 (18 to 37)
CCT (µm) (min, max values)	476.36 ± 43.25 (417, 612)
Average K value (D) (min, max values)	48.03 ± 4.32 (41.0, 61.0)
Maximum K value (D) (min, max values)	56.32 ± 8.81 (43.4, 82.4)

CCT = central corneal thickness; D = dioptre; max = maximum; K = keratometry; min = minimum. The data are shown as means ± standard deviation or minimum and maximum values.

**Table 2:** Changes in Visual Acuity and Corneal Morphology (*n* = 53) (mean ± SD).

Parameter	Preoperative	1-Mo Postoperative	LMM Change (95% CI)	*p*
UCVA (logMAR)	0.95 ± 0.47	0.96 ± 0.41	+0.0091 (−0.0854, 0.1035)	0.851
CDVA (logMAR)	0.31 ± 0.41	0.35 ± 0.40	+0.0479 (0.0071, 0.0888)	**0.** 022 *
K_max_ (D)	56.32 ± 8.81	57.66 ± 9.77	+1.3396 (0.5333, 2.1459)	0.001 **
**Anterior surface**				
K-flat (D)	46.65 ± 3.91	47.52 ± 4.62	+0.8717 (0.418, 1.3254)	**<** 0.001 **
K-steep (D)	49.68 ± 4.87	50.98 ± 5.74	+1.2962 (0.5686, 2.0238)	**<** 0.001 **
K-average (D)	48.03 ± 4.32	49.16 ± 5.05	+1.1283 (0.5697, 1.6869)	**<** 0.001 **
Astigmatism (D)	3.09 ± 2.13	3.24 ± 2.55	+0.1547 (−0.4911, 0.8006)	0.639
**Posterior surface**				
K-flat (D)	−6.91 ± 0.70	−6.97 ± 0.75	−0.0604 (−0.1368, 0.016)	0.121
K-steep (D)	−7.56 ± 0.95	−7.58 ± 0.90	−0.0189 (−0.1204, 0.0827)	0.716
K-average (D)	−7.22 ± 0.79	−6.99 ± 2.23	+0.2302 (−0.37, 0.8303)	0.452
Astigmatism (D)	0.64 ± 0.47	0.65 ± 0.40	+0.0151 (−0.0572, 0.0874)	0.682
**Apex point**				
Thickness (μm)	476.36 ± 43.25	464.38 ± 41.54	−11.9811 (−15.294, −8.6683)	**<** 0.001 **
Anterior elevation (μm)	18.17 ± 14.86	22.09 ± 18.77	+3.9245 (1.9976, 5.8515)	**<** 0.001 **
Posterior elevation (μm)	38.47 ± 27.91	40.58 ± 31.28	+2.1132 (−0.1669, 4.3933)	0.069
**Thinnest point**				
Thickness (μm)	471.06 ± 42.63	457.98 ± 39.65	−13.0755 (−17.5093, −8.6416)	**<** 0.001 **
Anterior elevation (μm)	24.75 ± 16.15	25.45 ± 17.77	+0.6981 (−2.7503, 4.1465)	0.692
Posterior elevation (μm)	50.75 ± 28.25	51.17 ± 30.80	+0.4151 (−1.8556, 2.6858)	0.720

UDVA = uncorrected distance visual acuity; CDVA = corrected distance visual acuity; K = keratometry; Kmax = maximum keratometry. *: *p* < 0.05, significant differences were observed (bold text) between the 1-month postoperative and preoperative values. **: *p* < 0.001, significant differences were observed between the 1-month postoperative and preoperative values. Re-analysis performed with a linear mixed-effects model (LMM), including a random intercept for patient (*N* = 53 eyes); pre- and postoperative means ± SD are from the same *n* = 53 dataset.

**Table 3:** Changes in Corneal Densitometry (*n* = 53) (mean ± SD).

Parameter	Preoperative	1-Mo Follow-Up	LMM Change (95% CI)	*p*
**0–2 mm annulus**				
Anterior (120 μm)	21.93 ± 4.55	33.54 ± 5.57	+11.6075 (10.3815, 12.8336)	**<** 0.001 **
Middle	13.98 ± 1.89	18.79 ± 3.58	+4.8122 (4.0138, 5.6106)	**<** 0.001 **
Posterior (60 μm)	8.79 ± 1.82	10.62 ± 2.40	+1.8285 (1.2987, 2.3583)	**<** 0.001 **
Total	15.05 ± 2.62	21.02 ± 3.47	+5.9667 (5.2069, 6.7264)	**<** 0.001 **
**2–6 mm annulus**				
Anterior (120 μm)	19.67 ± 3.47	27.73 ± 4.19	+8.0604 (7.1156, 9.0051)	**<** 0.001 **
Middle	12.39 ± 1.68	16.12 ± 3.16	+3.7264 (3.0179, 4.4349)	**<** 0.001 **
Posterior (60 μm)	9.56 ± 1.52	10.52 ± 1.66	+0.9528 (0.5147, 1.391)	**<** 0.001 **
Total	13.88 ± 2.02	18.12 ± 2.74	+4.2491 (3.6643, 4.8338)	**<** 0.001 **
**6–10 mm annulus**				
Anterior (120 μm)	18.15 ± 3.32	20.03 ± 3.05	+1.8792 (1.0368, 2.7217)	**<** 0.001 **
Middle	11.98 ± 1.78	12.58 ± 1.77	+0.5906 (0.1972, 0.9839)	0.003 *
Posterior (60 μm)	9.76 ± 1.23	9.92 ± 1.26	+0.1585 (−0.2123, 0.5293)	0.402
Total	13.33 ± 2.00	14.17 ± 1.92	+0.8409 (0.3283, 1.3535)	0.001 **
**10–12 mm annulus**				
Anterior (120 μm)	31.36 ± 12.40	30.51 ± 13.72	−0.8585 (−3.9556, 2.2386)	0.587
Middle	18.96 ± 7.76	19.18 ± 7.43	+0.217 (−1.5061, 1.9401)	0.805
Posterior (60 μm)	14.08 ± 4.98	14.65 ± 4.86	+0.566 (−0.5822, 1.7143)	0.334
Total	21.48 ± 7.65	21.68 ± 7.86	+0.1943 (−1.55, 1.9387)	0.827
**Total**				
Anterior (120 μm)	20.75 ± 3.01	26.27 ± 3.38	+5.5607 (4.7951, 6.3263)	**<** 0.001 **
Middle	13.40 ± 2.12	15.49 ± 1.98	+2.0943 (1.5374, 2.6513)	**<** 0.001 **
Posterior (60 μm)	9.95 ± 1.07	10.69 ± 1.25	+0.7439 (0.4183, 1.0694)	**<** 0.001 **
Total	14.63 ± 1.71	17.39 ± 2.11	+2.7528 (2.267, 3.2387)	**<** 0.001 **

Groups corresponding to different ranges of the corneal annulus are indicated in bold. *: *p* < 0.05, statistical differences existed (bold text) between the 1-month postoperative and preoperative levels. **: *p* < 0.001, significant differences existed between the 1-month postoperative and preoperative levels. Re-analysis performed with a linear mixed-effects model (LMM), including a random intercept for patient (*n* = 53 eyes); Pre/Post means ± SD are from the same *n* = 53 dataset.

**Table 4:** Correlation analysis and agreement between corneal densitometry and AS-OCT or IVCM.

Comparison	*n*	Reference Method (Mean ± SD, %)	Corneal Densitometry (Mean ± SD, %)	Mean Ratio (95% LoA)	ICC(2,1) (95% CI)	Pearson R
DL depth (AS-OCT)	50	56.59 ± 16.14	53.90 ± 16.55	0.938 (0.67 to 1.32)	0.874 (0.776–0.927)	0.884 (*p* < 0.001 **)
Reaction-initiating depth (IVCM)	53	30.66 ± 13.20	32.62 ± 13.29	1.075 (0.57 to 2.04)	0.822 (0.664–0.912)	0.828 (*p* < 0.001 **)
Reaction-ending depth (IVCM)	53	73.10 ± 15.35	77.47 ± 15.82	1.057 (0.81 to 1.37)	0.841 (0.702–0.903)	0.872 (*p* < 0.001 **)
Ending point to endothelium (IVCM)	53	26.90 ± 15.35	22.53 ± 15.82	0.811 (0.35 to 1.87)	0.841 (0.701–0.906)	0.872 (*p* < 0.001 **)

Values are expressed as a percentage of the corresponding full corneal thickness (mean ± SD). Ratio = corneal densitometry ÷ reference method corneal thickness. Mean ratio and 95% limits of agreement (LoA) are reported on the ratio scale; the LoA therefore widens asymmetrically. ICC(2,1) = two-way random-effects, absolute-agreement intraclass correlation (single measures); 95% CIs by non-parametric bootstrap. All Pearson correlation coefficients were significant at *p* < 0.001 (bold text). For the DL-depth comparison the sample was *n* = 50 because three eyes had no measurable DL on AS-OCT; **: *p* < 0.001.

## Data Availability

The original contributions presented in this study, including the examination images of the 53 patients, are included in the Supplementary Materials. The coordinate software developed and utilised in this study is currently under patent application; accordingly, the software and associated data are available on request from the corresponding author due to intellectual property restrictions.

## Supplementary Materials

The following supporting information can be downloaded at: https://www.mdpi.com/article/10.3390/jcm15218214/s1, Figure S1: How to read the coordinates of the graph using the coordinate software; Figure S2: The depth of DL measured by AS-OCT; Figure S3: Changes in Corneal Densitometry; Table S1: CXL Methods in this study; Table S2: Depth of the DL measured by three instruments; Table S3: Demarcation Line Measured by AS-OCT and the corneal densitometry graph.

## Abbreviations

CXL	Corneal collagen cross-linking
DL	Corneal stromal demarcation line
AS-OCT	Anterior segment optical coherence tomography
IVCM	In vivo confocal microscopy
Kmax	Maximum keratometry
K-steep	Steep keratometry
K-flat	Flat keratometry
K-avg	Average keratometry
UDVA	Uncorrected distance visual acuity
CDVA	Corrected distance visual acuity
SE	Spherical equivalent
CLR peaks	Cross-linking reaction peak(s)
