# Supplementary material for: A New Method for Demarcation Line Evaluation: The Corneal Densitometry Graph
Source: J Clin Med. 2026 Oct 22;15(21):8214. doi: 10.3390/jcm15218214 (PMC13764079; doi:10.3390/jcm15218214)
Supplement: Supplementary file 1 [file jcm-15-08214-s001.zip › Table S2.pdf]

Supplementary Table S2. *Depth of the DL measured by three instruments*

Number of eyes	AS-OCT		Corneal densitometry		Reaction initiating depth		Reaction ending depth	
	Visualization of the DL	Depth of the DL	Visualization of the DL	Depth of the DL	Corneal densitometry	IVCM	Corneal densitometry	IVCM
**1**	YES	52.44%	YES	62.85%	52.81%	50.19%	82.53%	73.58%
**2**	YES	73.65%	YES	78.31%	65.29%	63.60%	88.07%	83.80%
**3**	YES	62.60%	YES	52.38%	36.90%	19.54%	70.83%	72.80%
**4**	YES	68.48%	YES	50.34%	33.18%	27.92%	80.81%	77.29%
**5**	YES	52.43%	YES	61.67%	39.07%	41.36%	70.76%	59.55%
**6**	NO	-	YES	53.35%	34.69%	34.65%	72.49%	59.21%
**7**	NO	-	YES	10.74%	4.25%	7.56%	34.68%	42.00%
**8**	YES	16.21%	YES	10.30%	2.81%	2.17%	51.99%	43.48%
**9**	YES	27.36%	YES	37.92%	29.38%	33.75%	74.38%	79.17%
**10**	NO	-	YES	61.07%	42.01%	39.77%	23.16%	42.73%
**11**	YES	49.20%	YES	51.78%	41.09%	30.87%	90.26%	86.96%
**12**	YES	73.38%	YES	50.25%	9.56%	11.01%	83.09%	81.65%
**13**	YES	44.02%	YES	45.76%	26.91%	28.76%	83.18%	83.69%
**14**	YES	33.68%	YES	29.79%	20.91%	25.11%	52.69%	44.26%
**15**	YES	40.81%	YES	45.92%	27.64%	31.20%	72.54%	73.80%

**16**	YES	49.51%	YES	56.50%	32.39%	32.29%	86.52%	70.63%
**17**	YES	57.14%	YES	65.85%	25.00%	37.83%	84.15%	91.30%
**18**	YES	52.57%	YES	46.51%	23.26%	21.89%	77.52%	79.81%
**19**	YES	69.15%	YES	59.71%	53.23%	50.11%	70.89%	77.80%
**20**	YES	61.73%	YES	58.39%	33.09%	34.67%	85.89%	71.56%
**21**	YES	37.72%	YES	31.00%	17.90%	21.46%	70.31%	78.11%
**22**	YES	57.78%	YES	45.66%	25.83%	31.60%	69.63%	63.80%
**23**	YES	55.86%	YES	53.56%	31.11%	33.61%	76.00%	73.98%
**24**	YES	77.97%	YES	77.39%	58.25%	42.31%	93.08%	93.85%
**25**	YES	82.00%	YES	74.36%	52.42%	59.11%	92.38%	91.11%
**26**	YES	48.95%	YES	51.38%	37.79%	44.55%	74.19%	82.27%
**27**	YES	64.13%	YES	70.41%	46.78%	30.38%	91.41%	69.62%
**28**	YES	74.23%	YES	73.17%	31.91%	38.28%	80.28%	66.60%
**29**	YES	75.68%	YES	72.64%	57.34%	50.10%	89.54%	89.35%
**30**	YES	67.74%	YES	69.08%	40.13%	36.54%	87.06%	80.55%
**31**	YES	36.70%	YES	36.57%	31.38%	32.44%	76.07%	73.33%
**32**	YES	55.58%	YES	61.57%	28.94%	31.80%	92.13%	87.87%
**33**	YES	65.88%	YES	67.26%	45.52%	32.08%	85.43%	76.46%
**34**	YES	80.43%	YES	76.77%	44.87%	42.14%	90.89%	92.86%
**35**	YES	79.25%	YES	78.46%	38.46%	40.25%	86.81%	72.41%
**36**	YES	38.44%	YES	23.18%	17.50%	19.61%	82.50%	71.68%
**37**	YES	79.65%	YES	68.88%	41.88%	29.80%	95.42%	74.49%

**38**	YES	70.64%	YES	63.65%	46.37%	44.35%	74.85%	59.20%
**39**	YES	78.36%	YES	68.49%	45.66%	42.53%	79.95%	69.01%
**40**	YES	45.90%	YES	58.45%	23.91%	19.89%	91.79%	90.67%
**41**	YES	50.60%	YES	35.46%	15.54%	16.79%	79.48%	83.02%
**42**	YES	55.91%	YES	57.05%	33.41%	39.41%	80.69%	79.28%
**43**	YES	56.23%	YES	55.86%	24.31%	16.90%	64.61%	52.67%
**44**	YES	20.76%	YES	16.84%	12.21%	13.60%	33.89%	27.00%
**45**	YES	47.50%	YES	39.07%	19.43%	17.45%	52.54%	52.36%
**46**	YES	40.32%	YES	39.76%	28.43%	24.66%	91.25%	94.72%
**47**	YES	53.65%	YES	45.01%	30.14%	27.25%	90.22%	95.49%
**48**	YES	74.53%	YES	74.58%	30.30%	8.50%	83.84%	75.17%
**49**	YES	66.78%	YES	59.47%	37.38%	9.49%	90.03%	73.79%
**50**	YES	65.44%	YES	55.46%	38.33%	28.21%	85.65%	81.47%
**51**	YES	51.81%	YES	54.05%	27.03%	30.00%	88.06%	83.13%
**52**	YES	56.78%	YES	58.09%	48.34%	52.83%	89.58%	84.07%
**53**	YES	33.91%	YES	22.68%	13.15%	10.22%	54.88%	50.22%

Here is a portion of the raw data showing 53 keratoconus eyes visibility and depth of DL 1 month after CXL (measured by three instruments)

** The blue list is the visibility and depth of DL from AS-OCT;*

The yellow list is the visibility and depth of DL from the Max corneal densitometry graph;

The red list is the depth of cross-linking reaction-initiating measured by the Max corneal densitometry graph and IVCM;

The green list is the depth of cross-linking reaction-ending measured by the Max corneal densitometry graph and IVCM;

** Percentage (%) represents the ratio of DL depth to the corresponding corneal thickness.*
