# Supplementary material for: A New Method for Demarcation Line Evaluation: The Corneal Densitometry Graph
Source: J Clin Med. 2026 Oct 22;15(21):8214. doi: 10.3390/jcm15218214 (PMC13764079; doi:10.3390/jcm15218214)
Supplement: Supplementary file 1 [file jcm-15-08214-s001.zip › Table S1.pdf]

Supplementary Table S1. *CXL Methods in this study*

Parameter	Surgical Procedures	Alternative Examples
Treatment target	Keratoconus, Ectasia	Infectious keratitis, therapeutic refractive, prophylaxis, others
Fluence (total) (J/cm^2)	5.4	7.2, 2.7, others
Soak time and interval (minutes)	30(q2)	10(q2), 20(q5), others
Intensity (mW)	9	3,5, 10, 14, 18, 30, others
Treatment time (minutes)	10	30, 9, 5, 4, 3, others
Epithelium status	Off (20% alcohol)	On, partial removal, iontophoresis, others
Chromophore	Riboflavin (VibeX Rapid, Avedro)	Rose Bengal, others
Chromophore carrier	Hydroxypropyl methylcellulose (HPMC)	Dextran, others
Chromophore osmolarity	Iso-osmolar	Hypo-osmolar, others
Chromophore concentration	0.1%	0.2%, others
Light source	370-nm UVA light (Avedro System)	-
Irradiation mode (interval)	Continuous	Pulsed (1 minute), others
Protocol modifications	Contact lens–assisted	Penetration enhancers (list details), others
Protocol abbreviation in manuscript	A-CXL(10*9) (Accelerated)	S-CXL(3*30) (Standard), others
