# Supplementary material for: A New Method for Demarcation Line Evaluation: The Corneal Densitometry Graph
Source: J Clin Med. 2026 Oct 22;15(21):8214. doi: 10.3390/jcm15218214 (PMC13764079; doi:10.3390/jcm15218214)
Supplement: Supplementary file 1 [file jcm-15-08214-s001.zip › Table S3.pdf]

Supplementary Table S3. *Demarcation Line Measured by AS-OCT and the corneal densitometry graph*

<table>
<tr><th>EYES</th><th colspan="3">AS-OCT</th><th colspan="3">Corneal densitometry graph</th></tr>
<tr><td>Depth</td><td>D.L.</td><td>Corneal thickness</td><td>%</td><td>D.L.</td><td>Corneal vertex thickness</td><td>%</td></tr>
<tr><td rowspan="2">1</td><td colspan="3">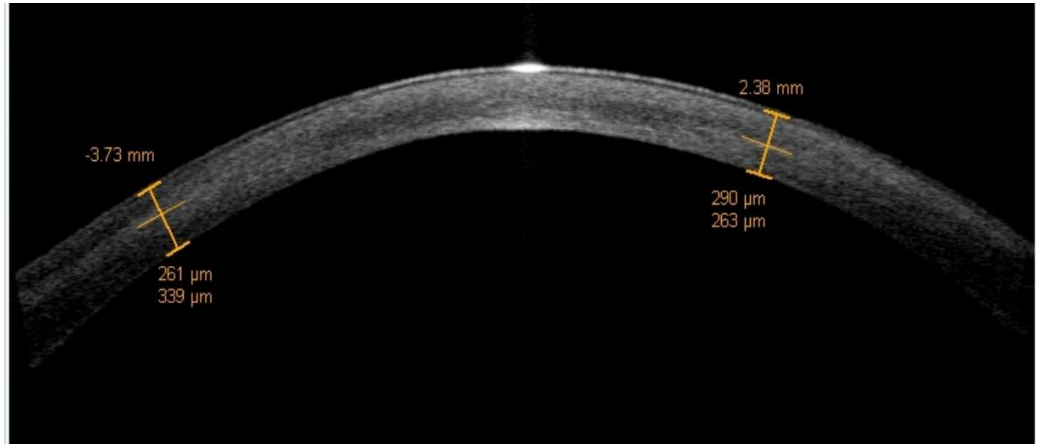
</td><td colspan="3">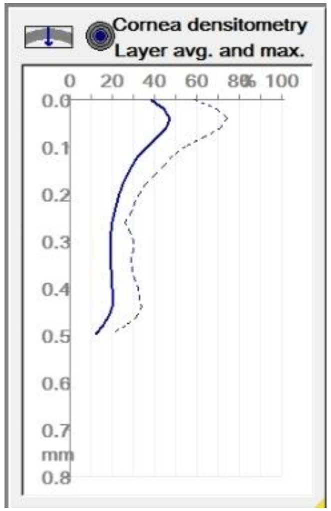
</td></tr>
<tr><td>290</td><td>553</td><td>52.44%</td><td>313</td><td>498</td><td>62.85%</td></tr>
</table>

2	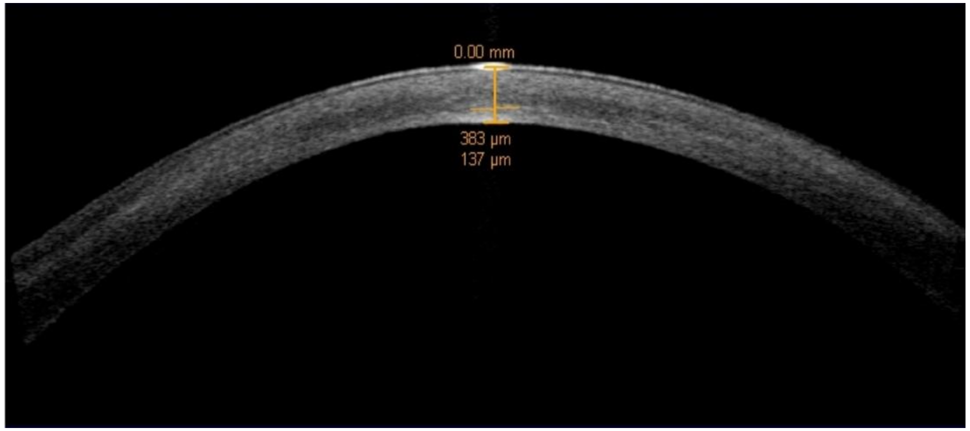			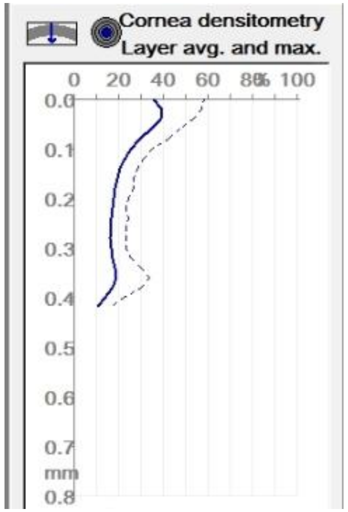		
	383	520	**73.65%**	361	461	**78.31%**
3	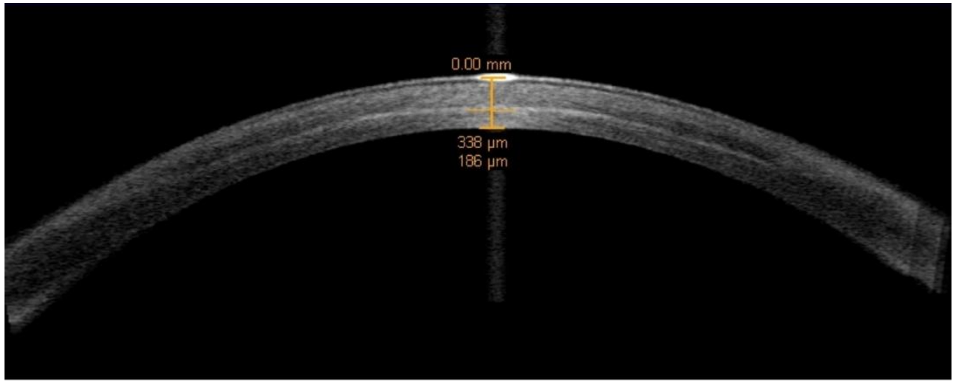			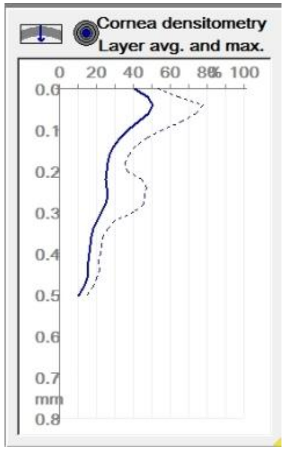		

	328	524	**62.60%**	264	504	**52.38%**
4	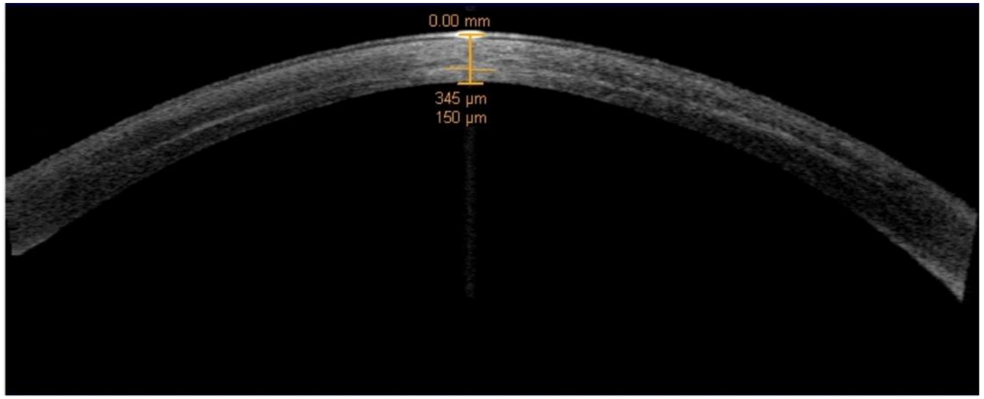			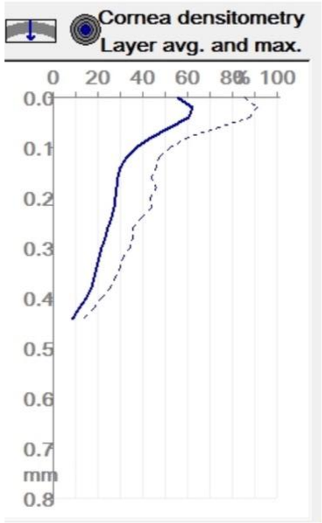		
	339	495	**68.48%**	223	443	**50.34%**

<table>
<tr><td rowspan="2">5</td><td colspan="3">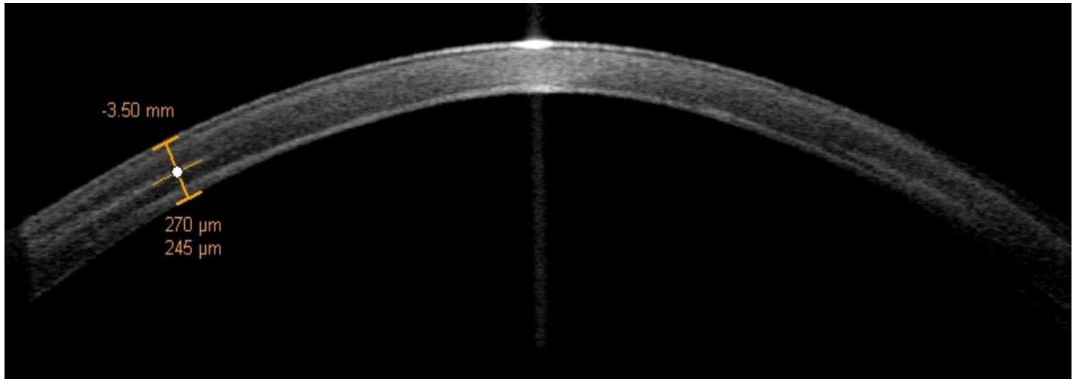
</td><td colspan="3">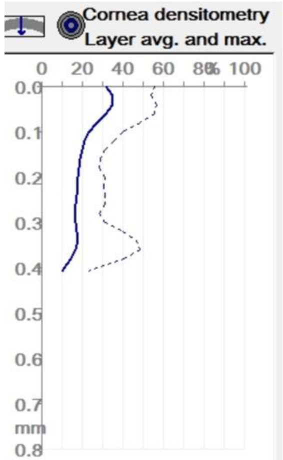
</td></tr>
<tr><td>270</td><td>515</td><td>52.43%</td><td>251</td><td>407</td><td>61.67%</td></tr>
<tr><td>6</td><td colspan="3">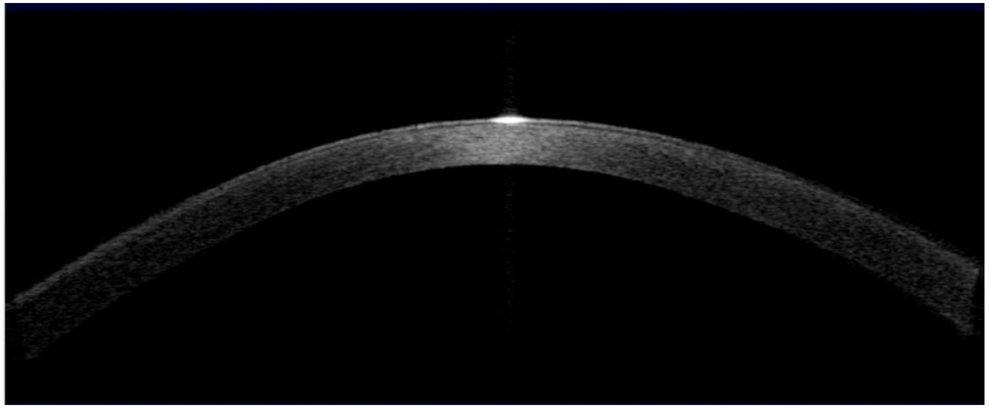</td><td colspan="3">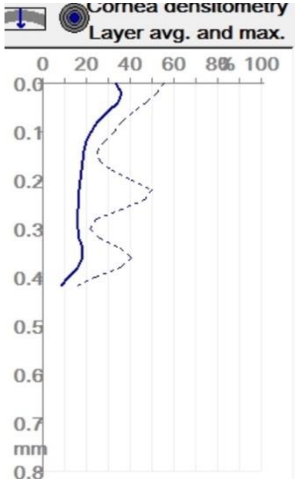
</td></tr>
</table>

	-	-	-	223	418	**53.35%**
7	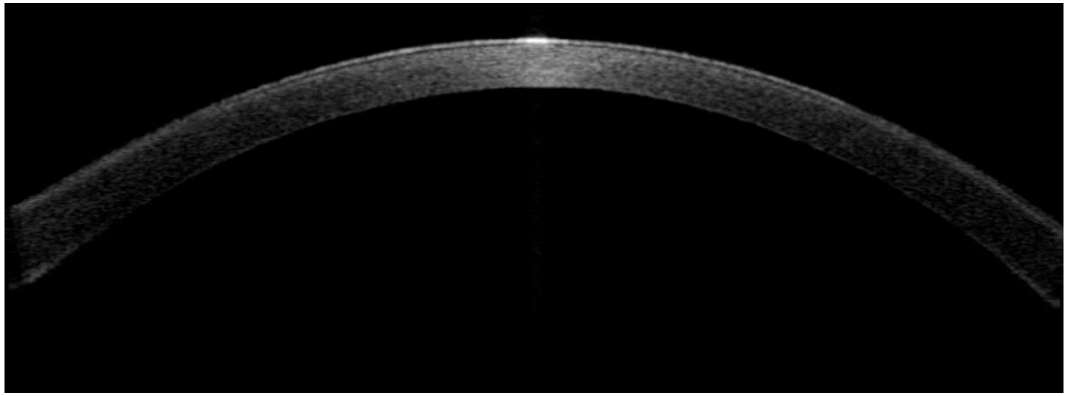			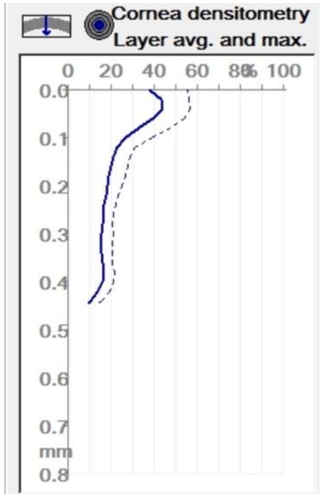		
	-	-	-	48	447	**10.74%**

<table>
<tr><td rowspan="2">8</td><td colspan="3">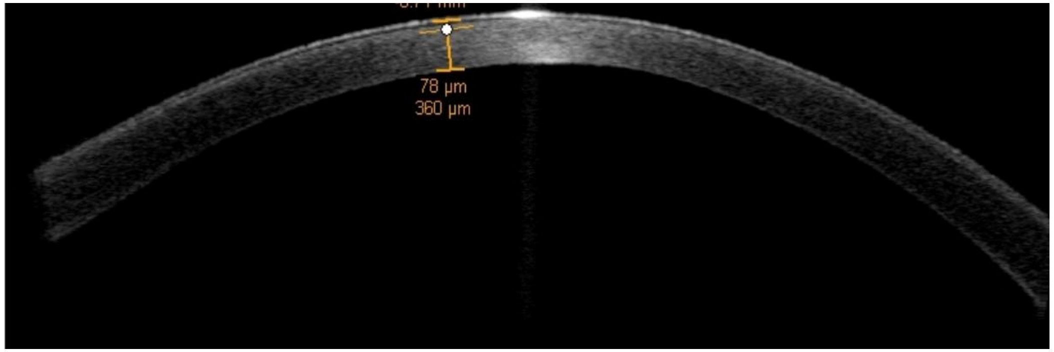
</td><td colspan="3">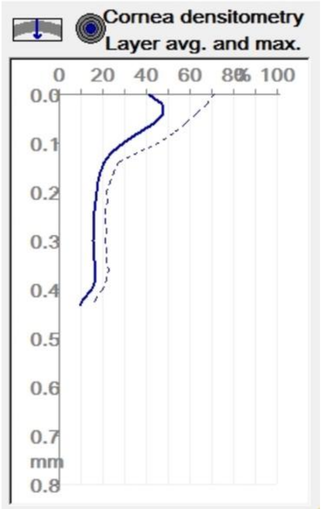
</td></tr>
<tr><td>71</td><td>438</td><td>16.21%</td><td>44</td><td>427</td><td>10.30%</td></tr>
<tr><td rowspan="2">9</td><td colspan="3">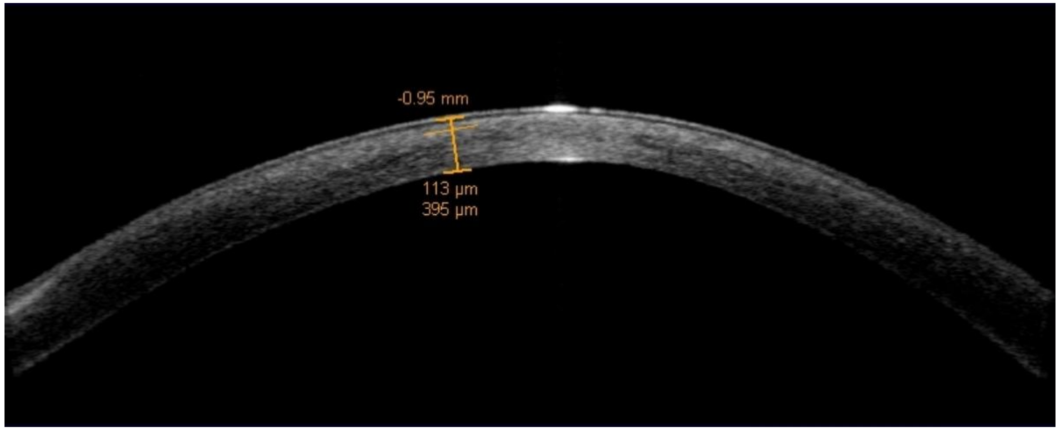
</td><td colspan="3">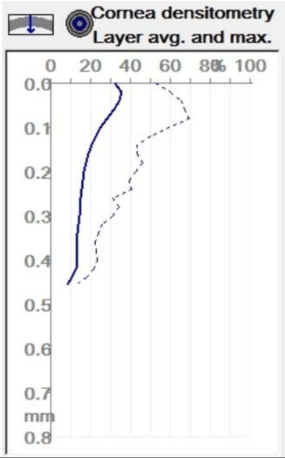
</td></tr>
<tr><td>139</td><td>508</td><td>27.36%</td><td>182</td><td>480</td><td>37.92%</td></tr>
</table>

<table>
<tr><td rowspan="2">10</td><td colspan="3">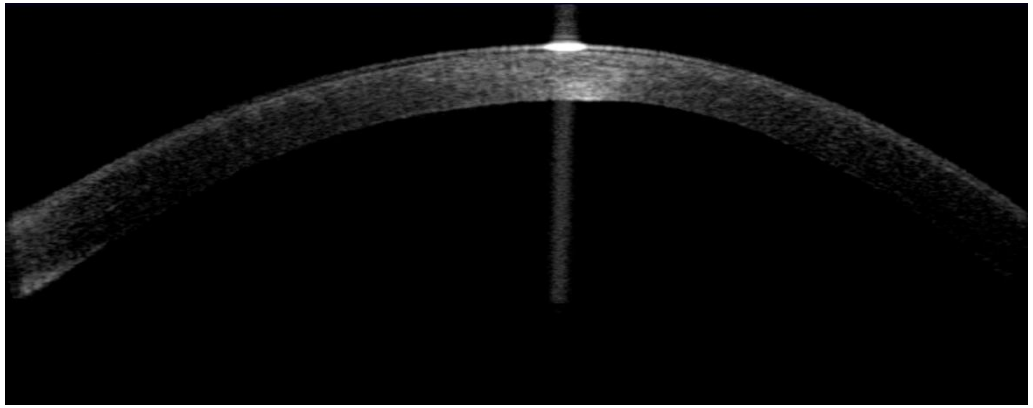</td><td colspan="3">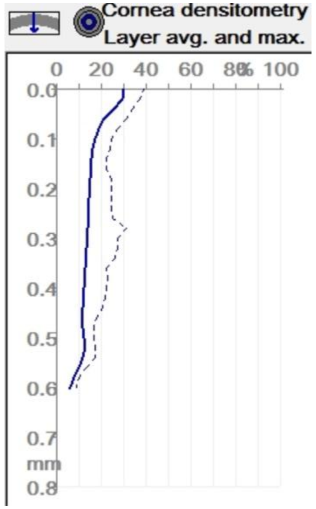</td></tr>
<tr><td>-</td><td>-</td><td>-</td><td>298</td><td>488</td><td>61.07%</td></tr>
<tr><td>11</td><td colspan="3">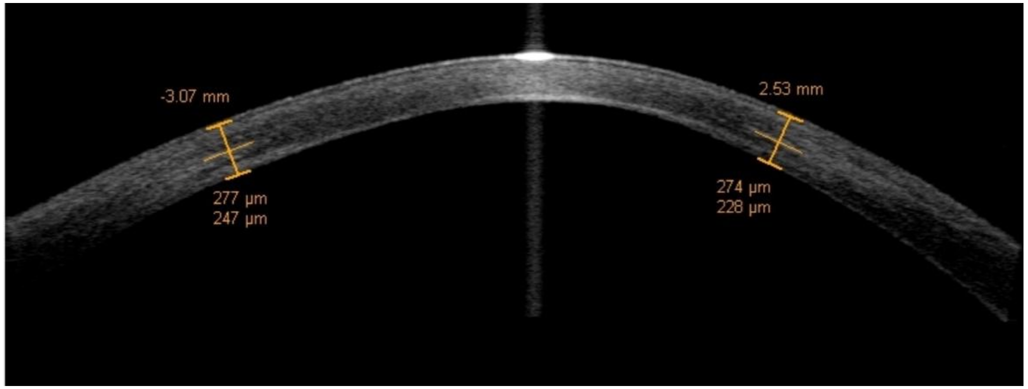</td><td colspan="3">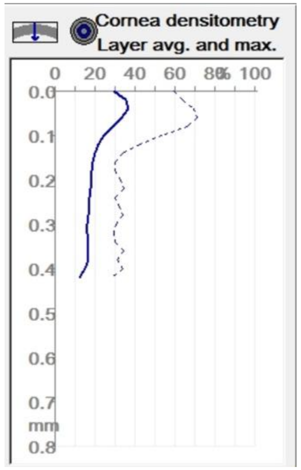</td></tr>
</table>

	247	502	**49.20%**	218	421	**51.78%**
12	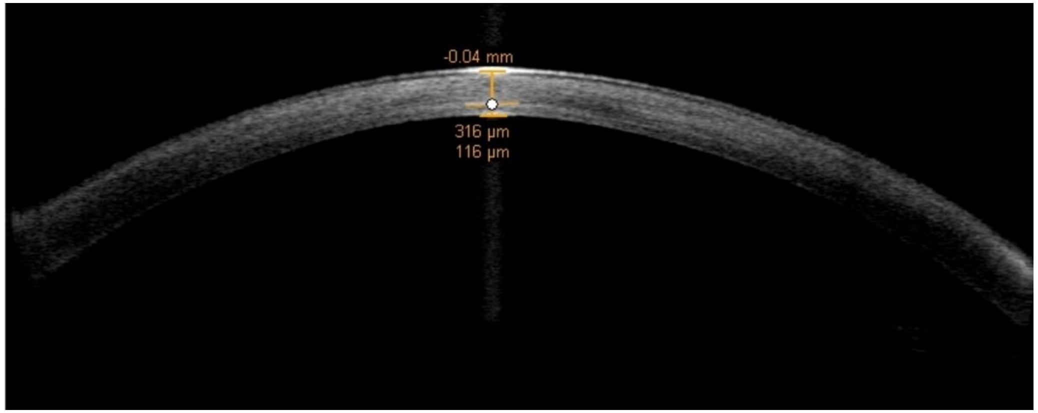			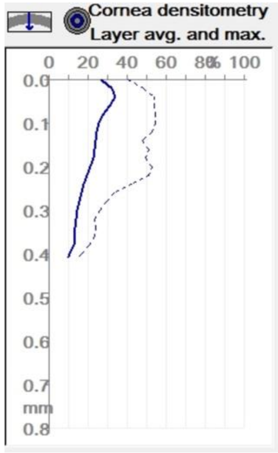		
	317	432	**73.38%**	205	408	**50.25%**
13	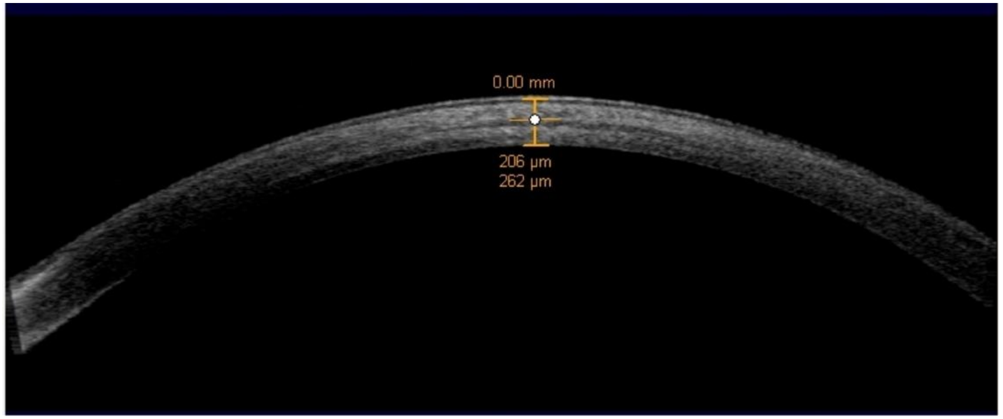			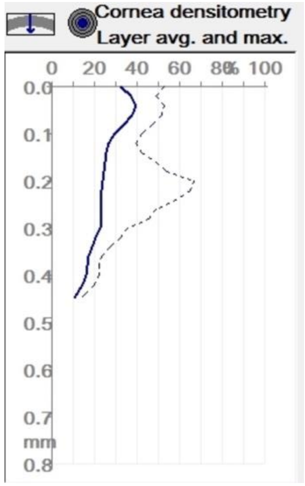		

<table>
<tr><td></td><td>206</td><td>468</td><td>44.02%</td><td>204.55</td><td>447</td><td>45.76%</td></tr>
<tr><td rowspan="2">14</td><td colspan="3">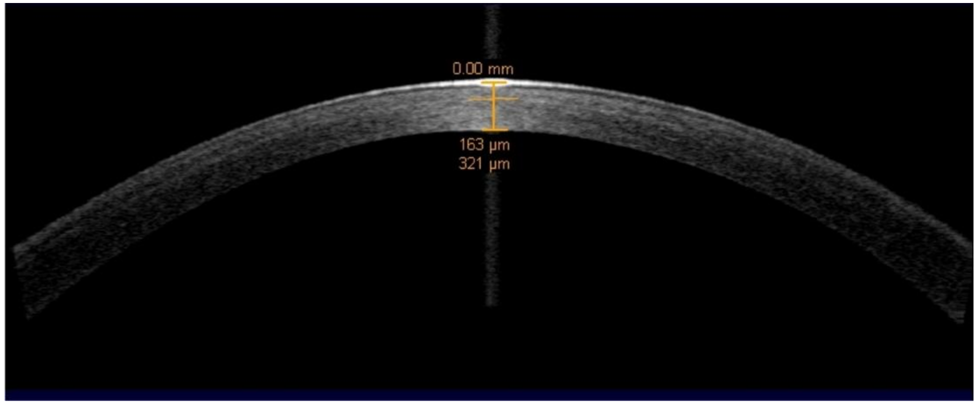
</td><td colspan="3">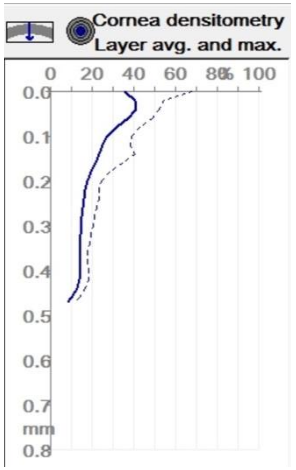
</td></tr>
<tr><td>163</td><td>484</td><td>33.68%</td><td>140.3</td><td>471</td><td>29.79%</td></tr>
<tr><td>15</td><td colspan="3">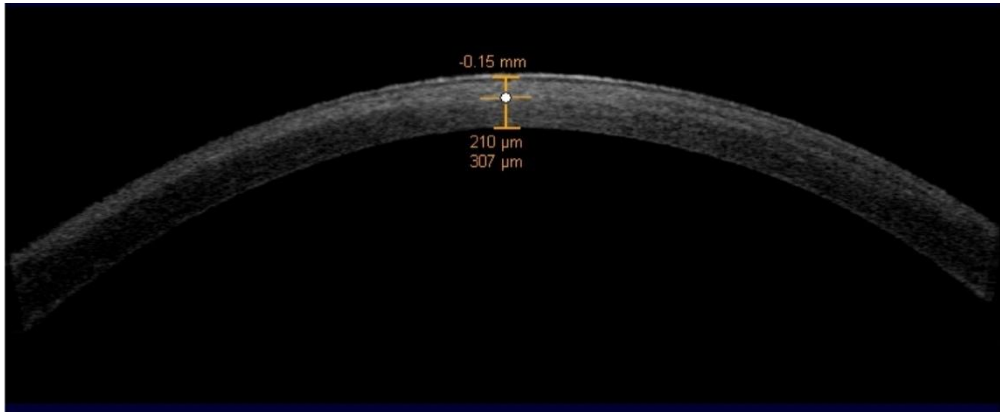
</td><td colspan="3">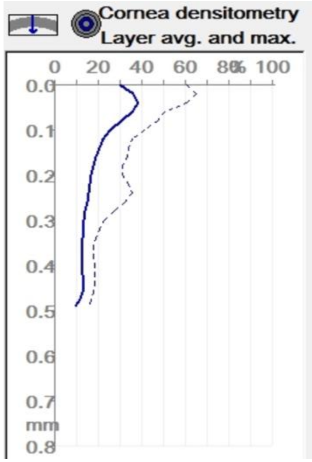
</td></tr>
</table>

	211	517	**40.81%**	225	490	**45.92%**
16	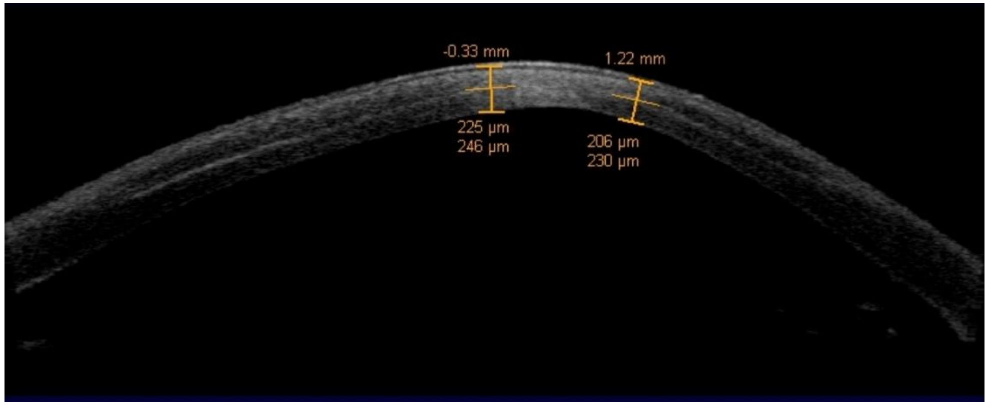			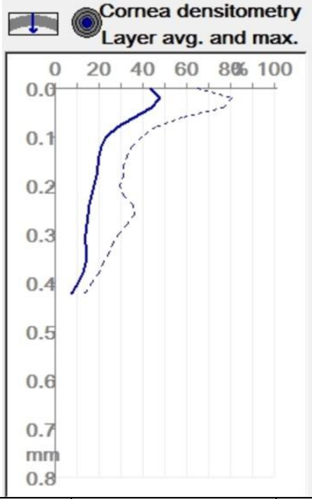		
	253	511	**49.51%**	239	423	**56.50%**
17	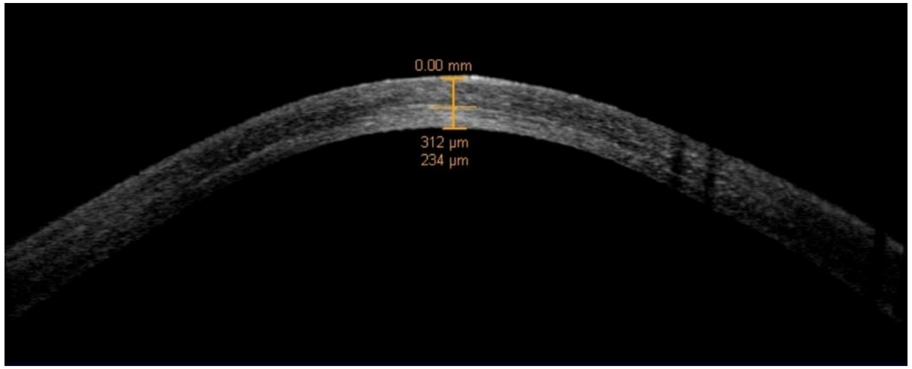			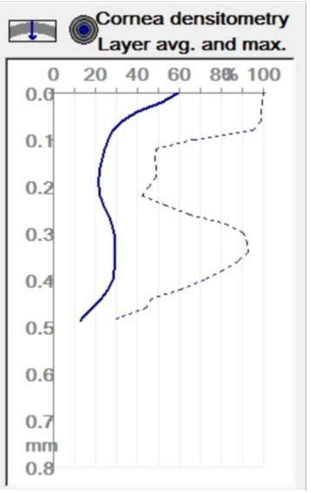		

	312	546	**57.14%**	324	492	**65.85%**
18	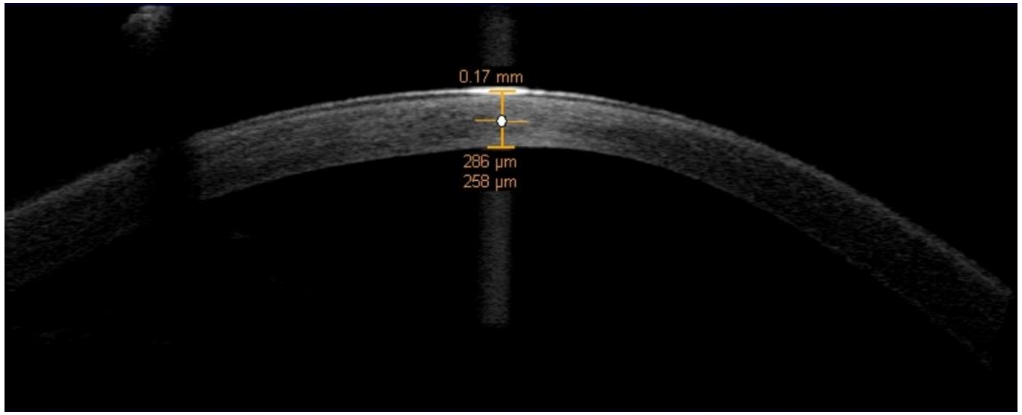			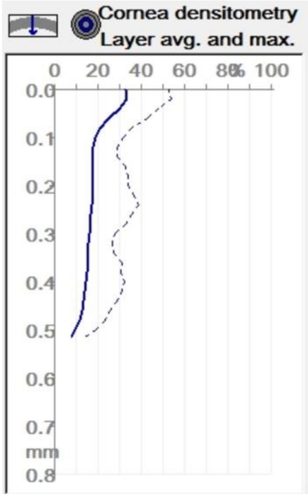		
	286	544	**52.57%**	240	516	**46.51%**
19	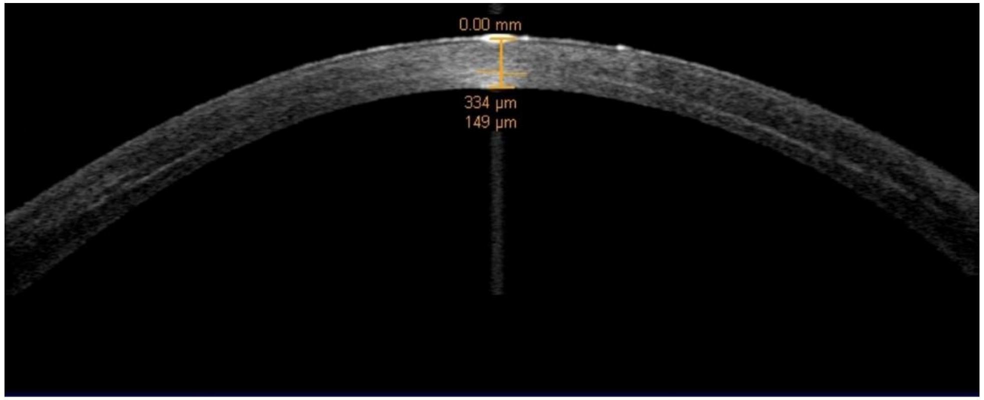			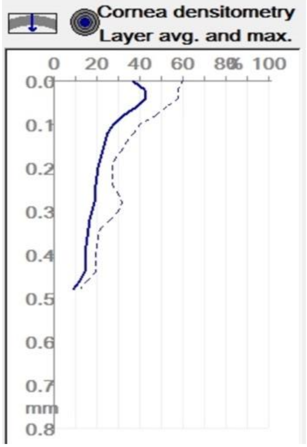		

<table>
<tr><td></td><td>324</td><td>483</td><td>67.08%</td><td>286</td><td>479</td><td>59.71%</td></tr>
<tr><td rowspan="2">20</td><td colspan="3">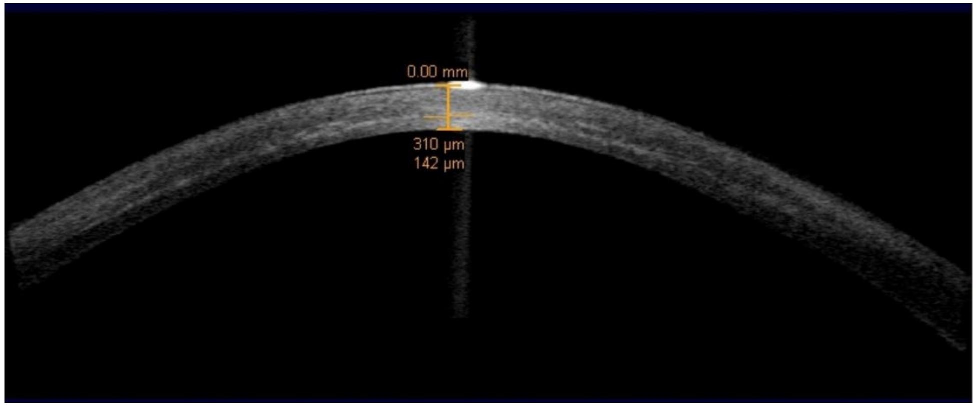
</td><td colspan="3">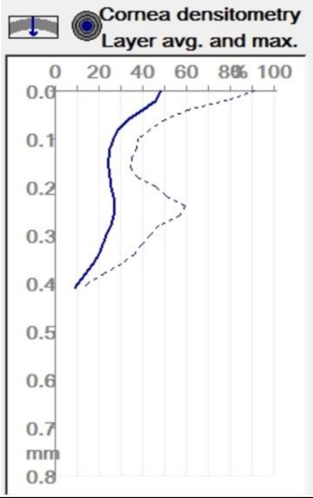
</td></tr>
<tr><td>279</td><td>452</td><td>61.73%</td><td>240</td><td>411</td><td>58.39%</td></tr>
<tr><td>21</td><td colspan="3">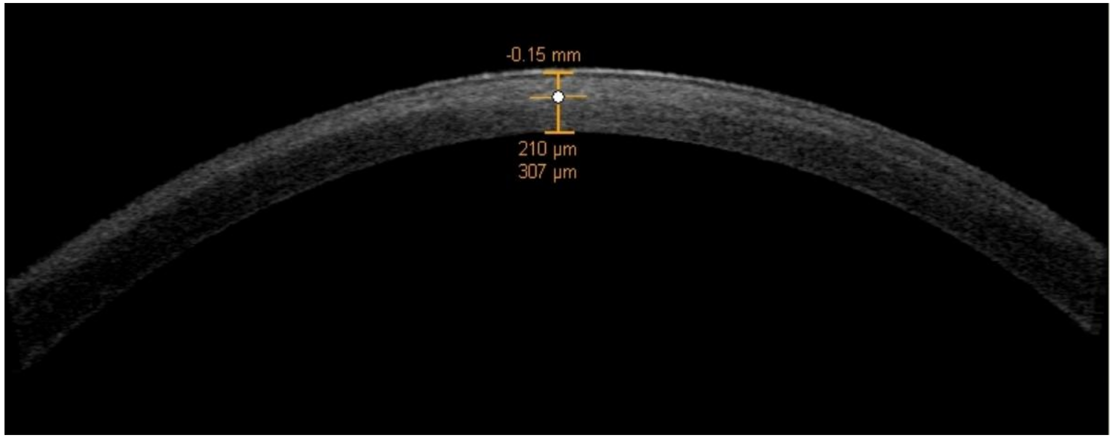
</td><td colspan="3">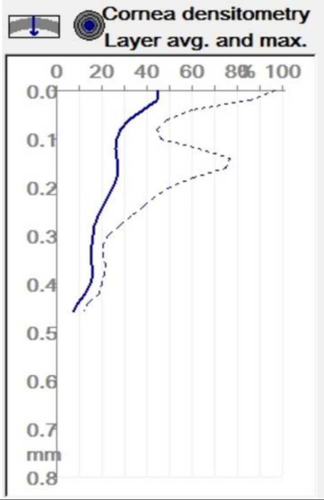
</td></tr>
</table>

	195	517	**37.72%**	142	458	**31.00%**
22	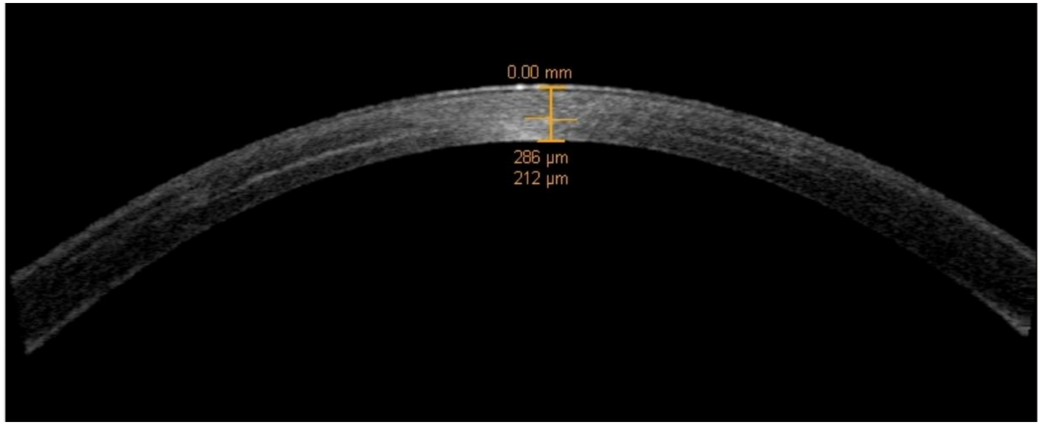			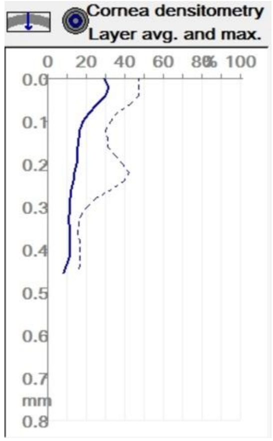		
	286	495	**57.78%**	221	484	**45.66%**
23	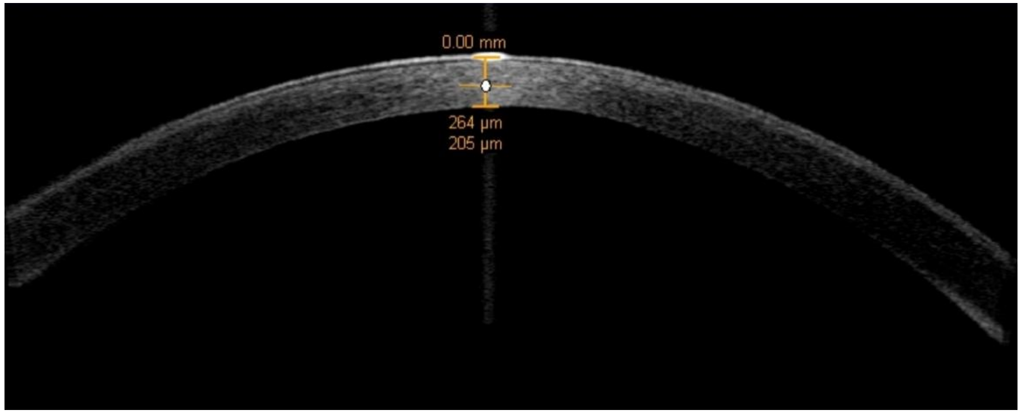			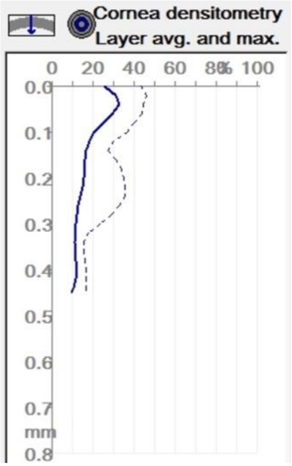		

	262	469	**55.86%**	241	450	**53.56%**
24	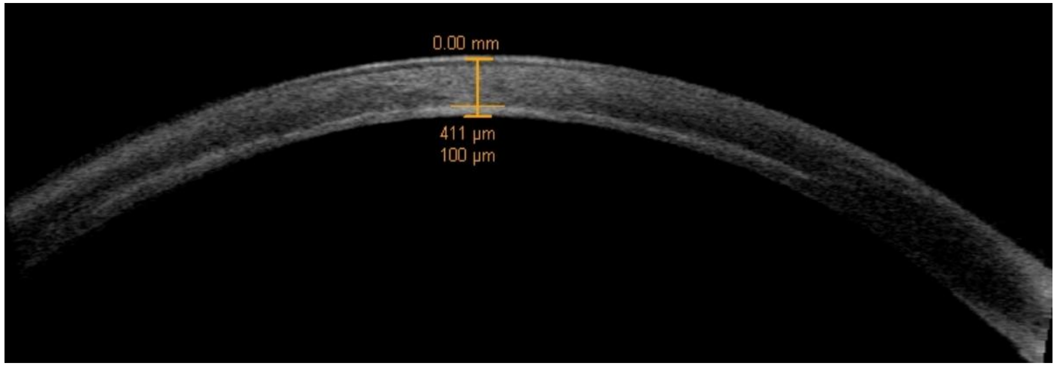			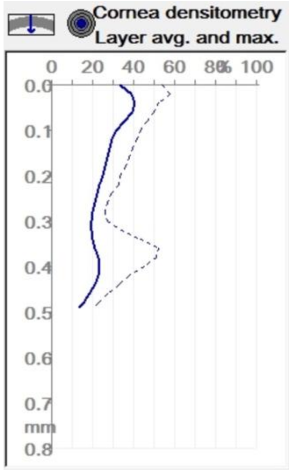		
	400	513	**77.97%**	380	491	**77.39%**
25	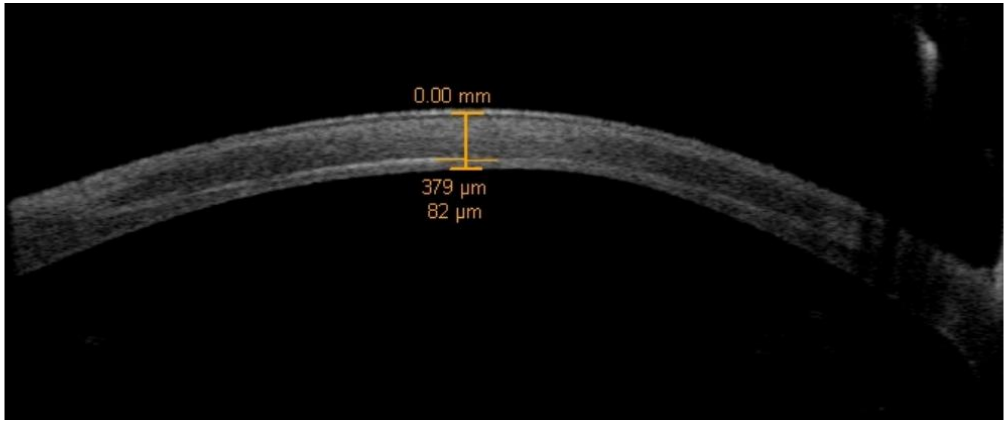			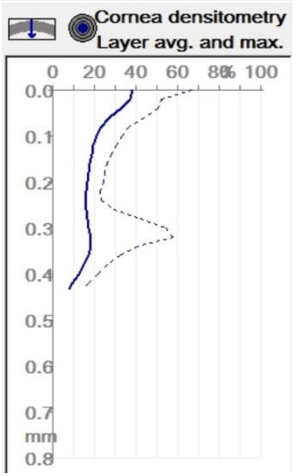		

<table>
<tr><td></td><td>378</td><td>461</td><td>82.00%</td><td>322</td><td>433</td><td>74.36%</td></tr>
<tr><td rowspan="2">26</td><td colspan="3">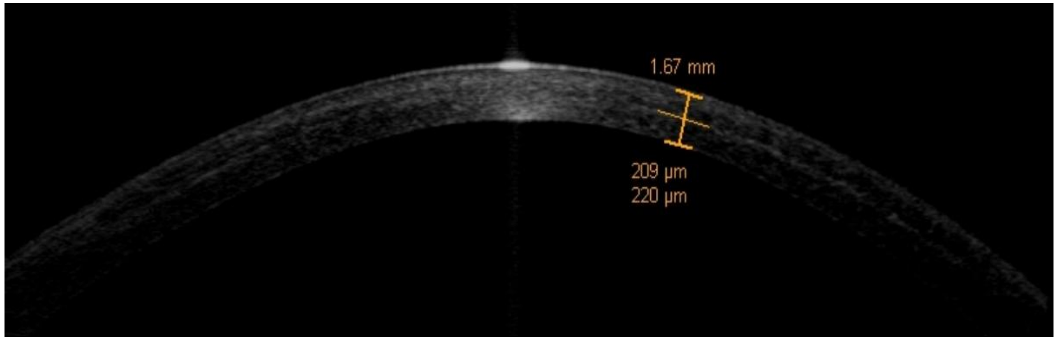
</td><td colspan="3">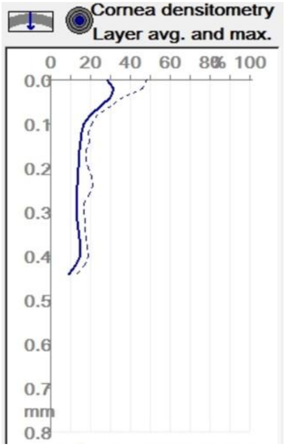
</td></tr>
<tr><td>210</td><td>429</td><td>48.95%</td><td>223</td><td>434</td><td>51.38%</td></tr>
<tr><td>27</td><td colspan="3">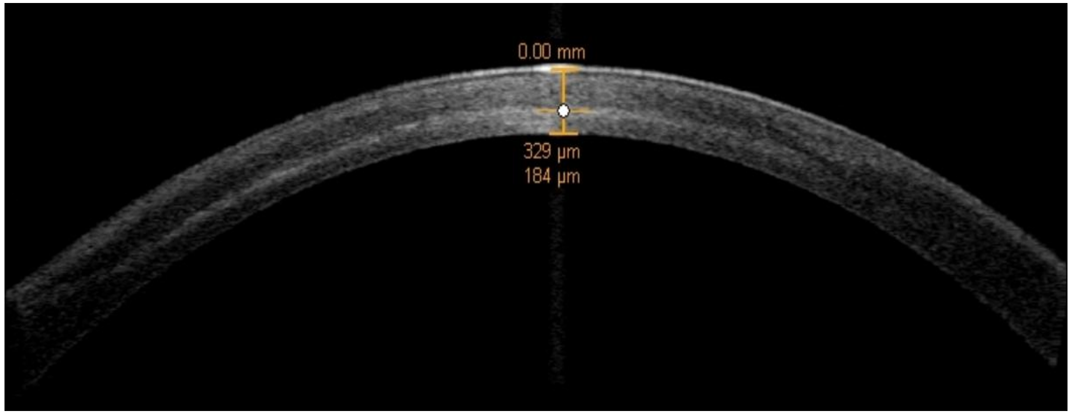
</td><td colspan="3">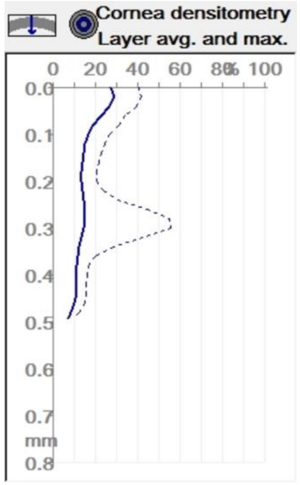
</td></tr>
</table>

	329	513	**64.13%**	295	419	**70.41%**
28	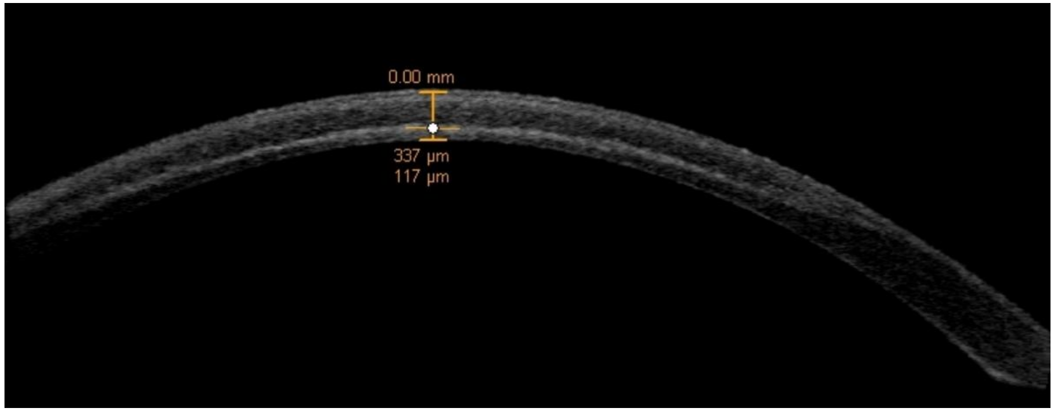			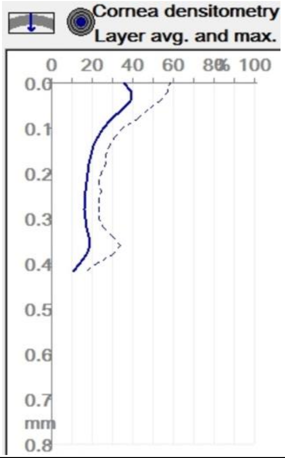		
	337	454	**74.23%**	360	492	**73.17%**
29	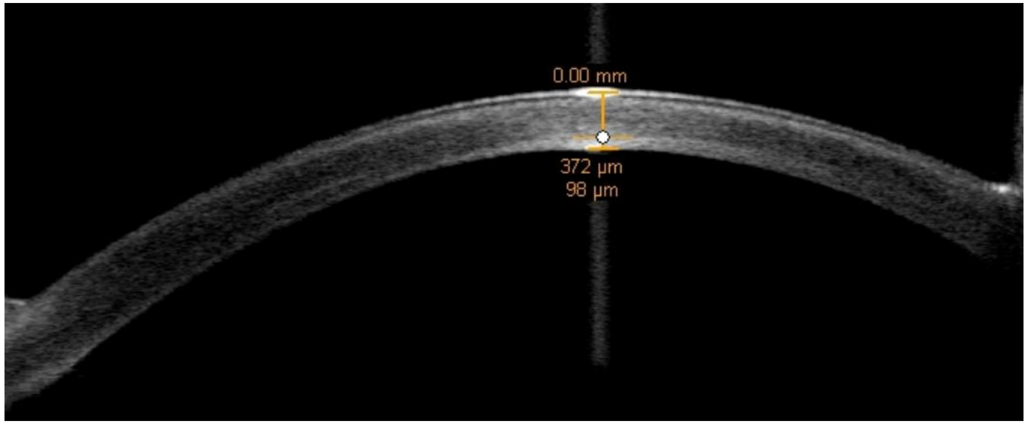			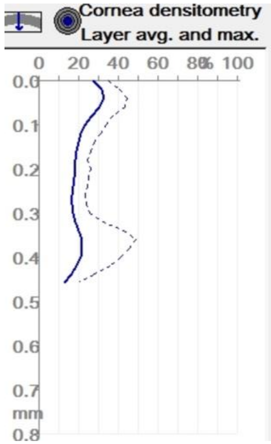		
	361	477	**75.68%**	361	497	**72.64%**

30	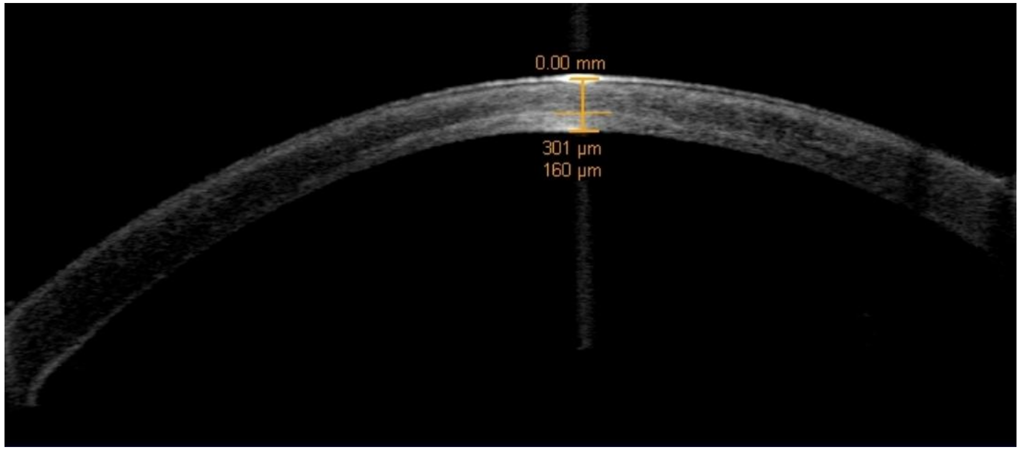			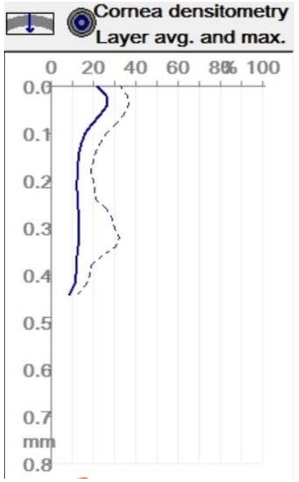		
	315	465	**67.74%**	315	456	**69.08%**
31	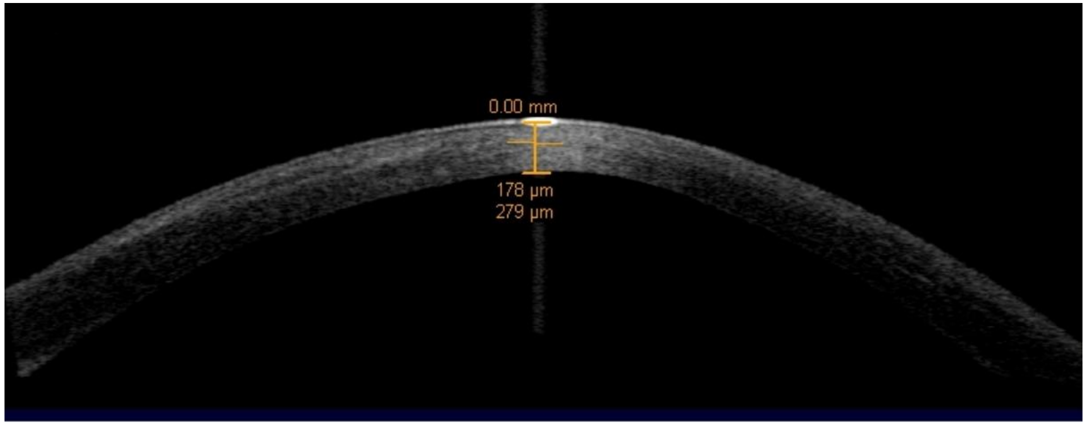			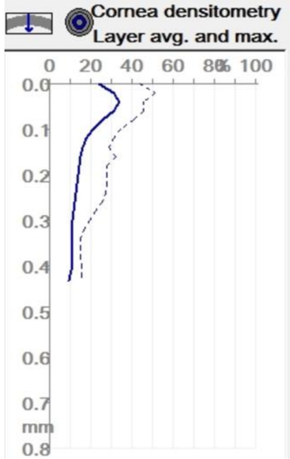		

	178	485	**36.70%**	162	443	**36.57%**
32	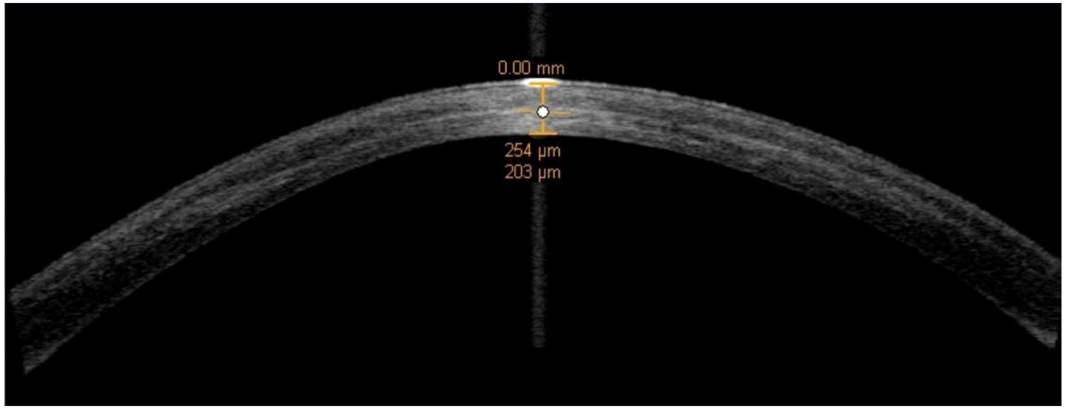			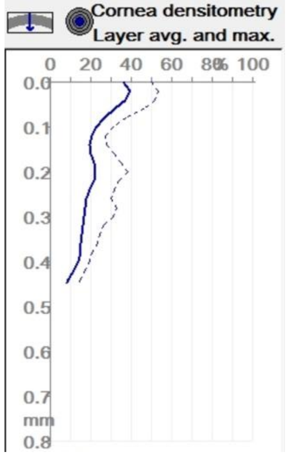		
	254	457	**55.58%**	266	432	**61.57%**
33	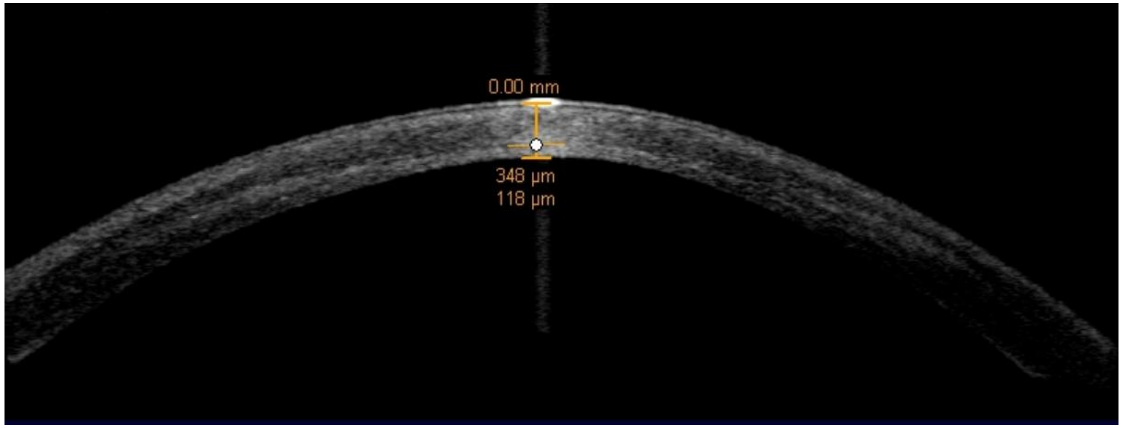			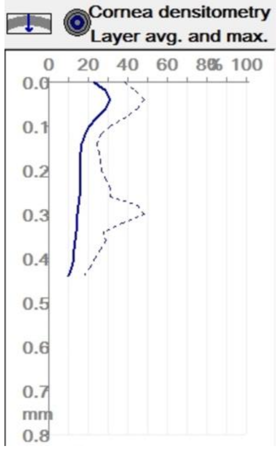		
	307	466	**65.88%**	300	446	**67.26%**

<table>
<tr><td rowspan="2">34</td><td colspan="3">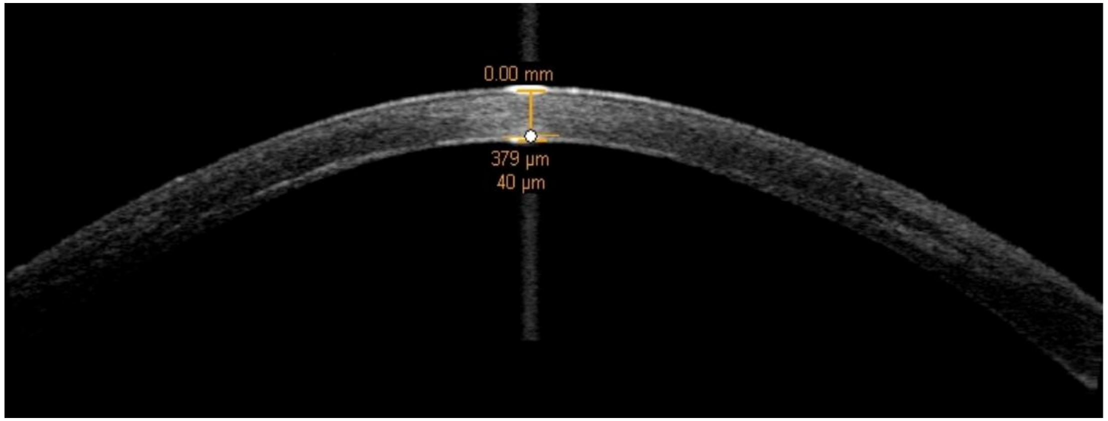
</td><td colspan="3">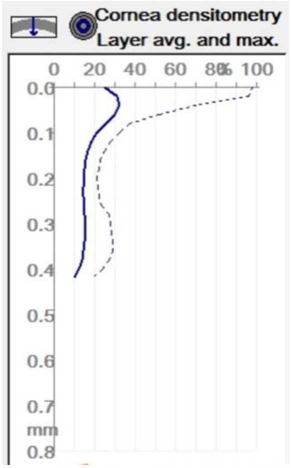
</td></tr>
<tr><td>337</td><td>419</td><td>80.43%</td><td>337</td><td>439</td><td>76.77%</td></tr>
<tr><td>35</td><td colspan="3">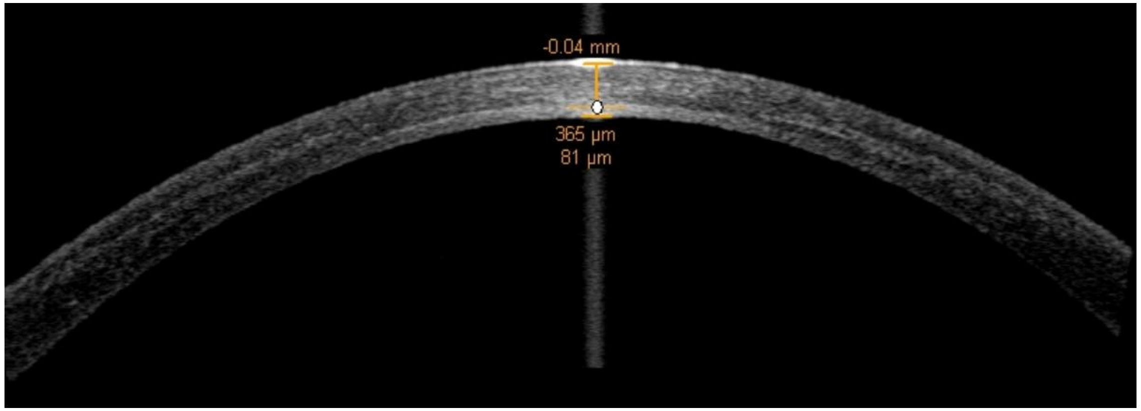
</td><td colspan="3">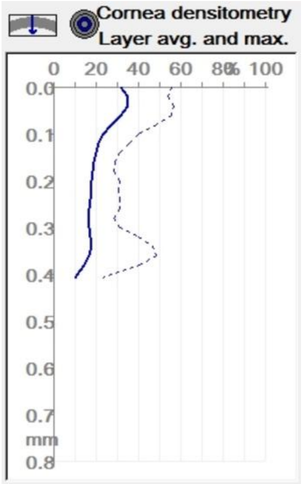
</td></tr>
</table>

	359	453	**79.25%**	357	455	**78.46%**
36	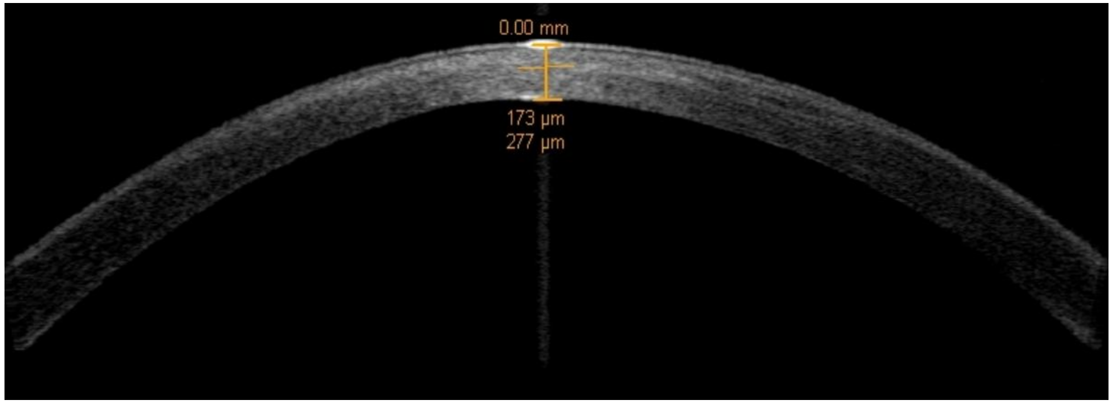			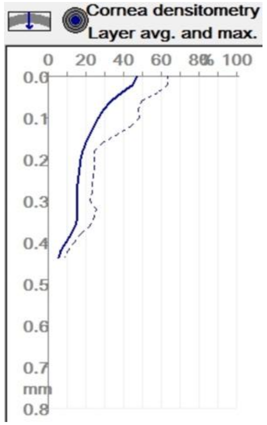		
	173	450	**38.44%**	102	440	**23.18%**
37	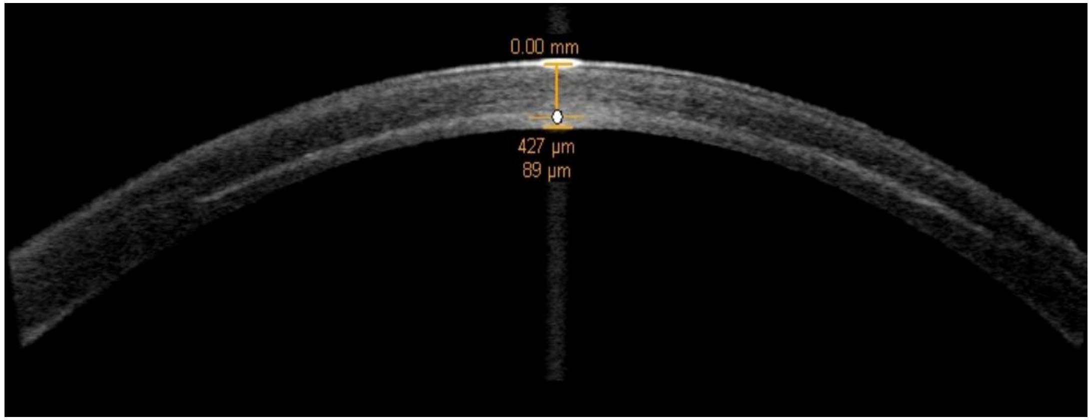			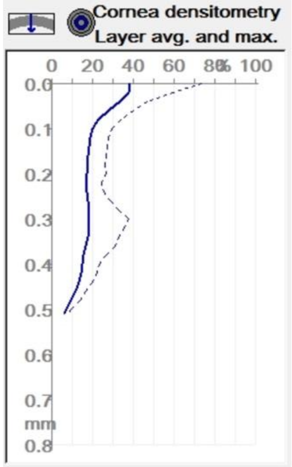		

	411	516	**79.65%**	301	437	**68.88%**
38	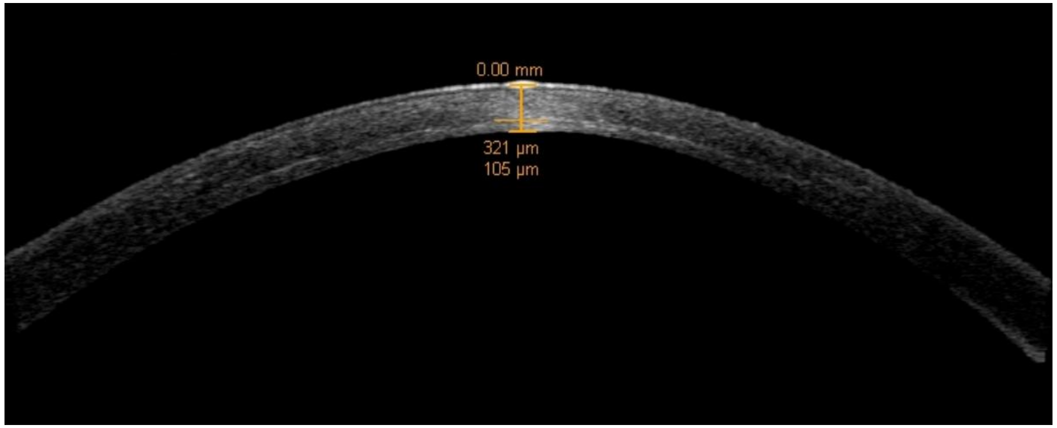			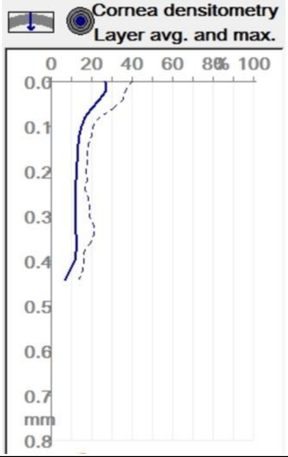		
	320	453	**70.64%**	324	509	**63.65%**
39	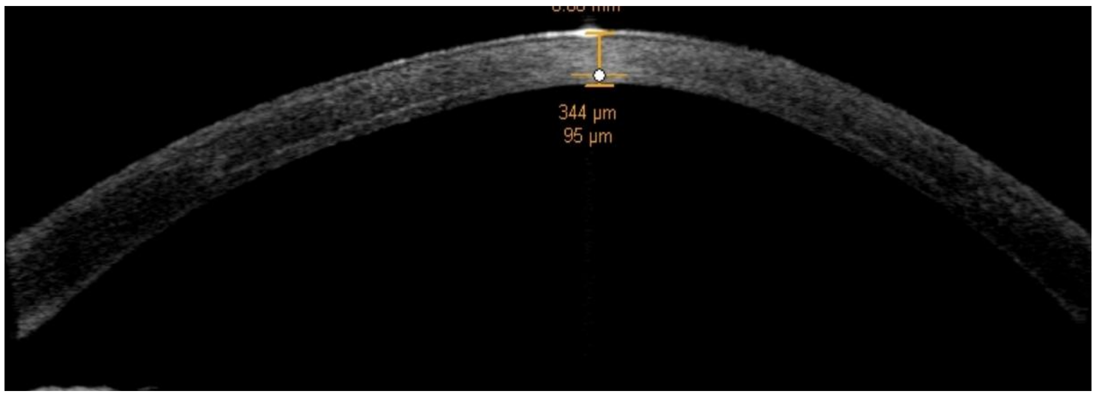			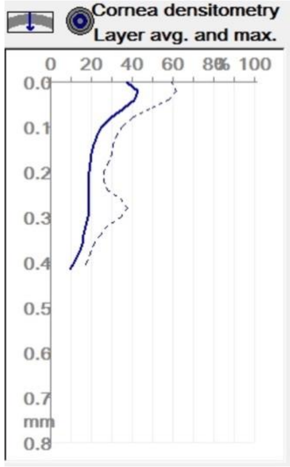		

<table>
<tr><td></td><td>344</td><td>439</td><td>78.36%</td><td>281</td><td>438</td><td>64.16%</td></tr>
<tr><td rowspan="2">40</td><td colspan="3">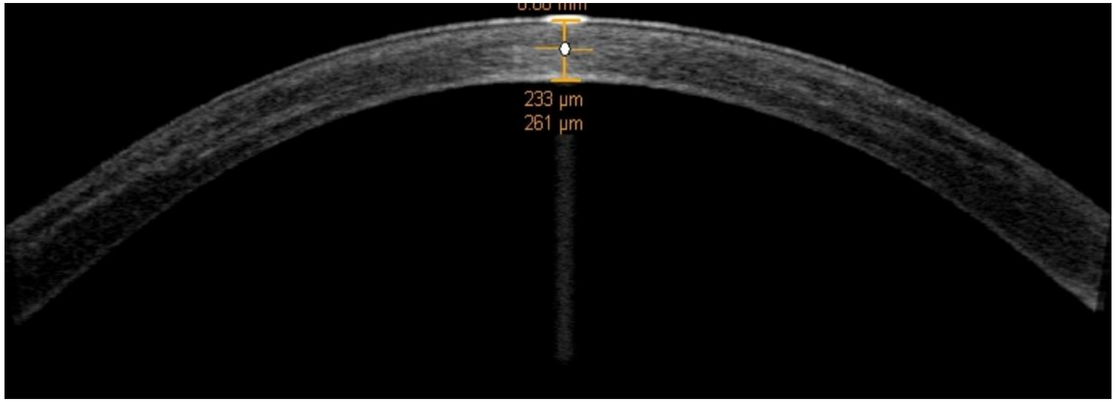
</td><td colspan="3">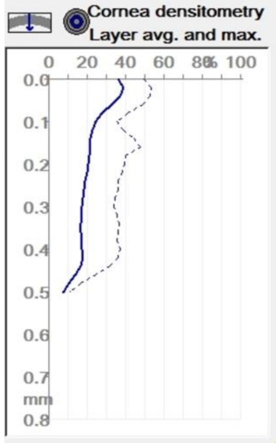
</td></tr>
<tr><td>224</td><td>488</td><td>45.90%</td><td>242</td><td>414</td><td>58.45%</td></tr>
<tr><td>41</td><td colspan="3">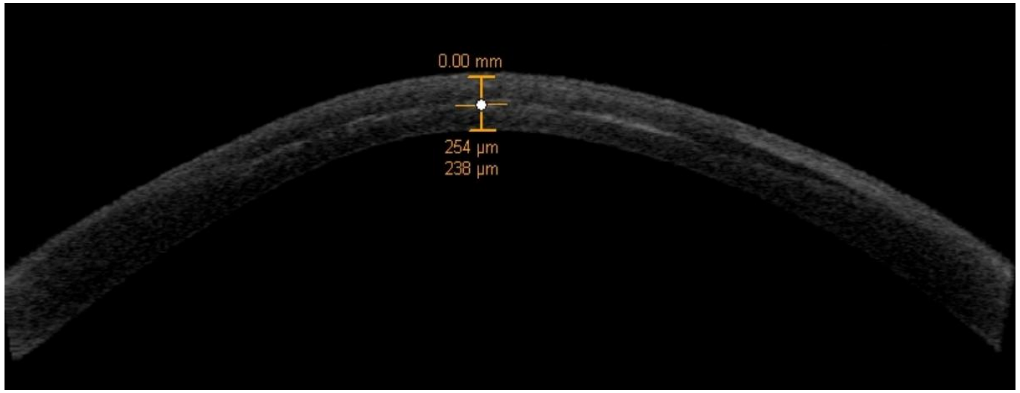
</td><td colspan="3">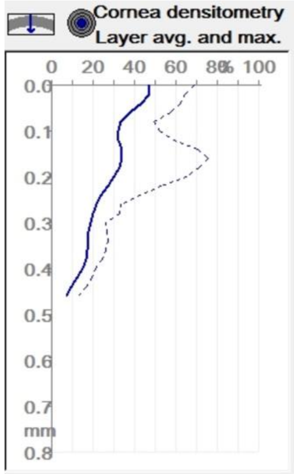
</td></tr>
</table>

	253	500	**50.60%**	178	502	**35.46%**
42	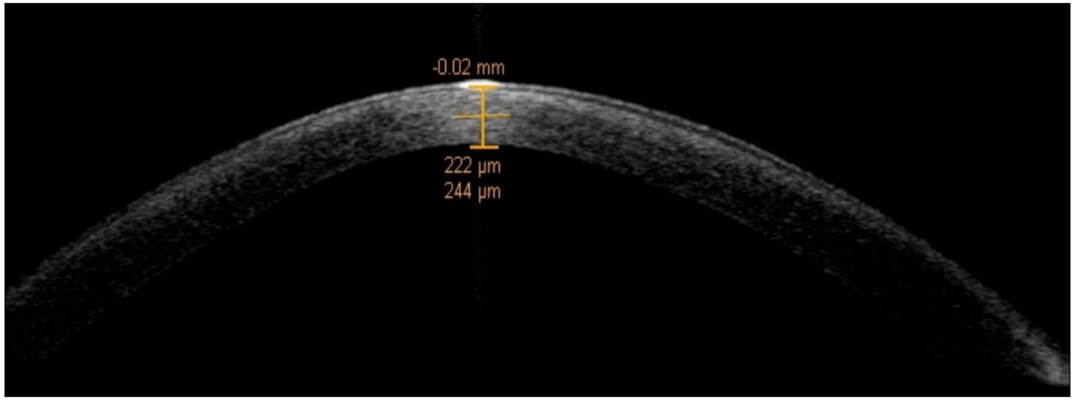			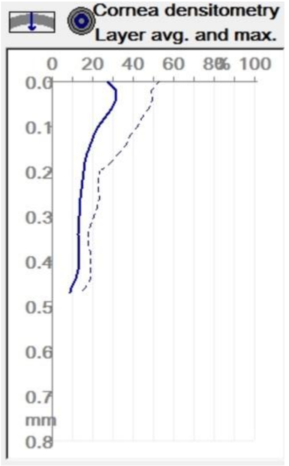		
	260	465	**55.91%**	263	461	**57.05%**

<table>
<tr><td rowspan="2">43</td><td colspan="3">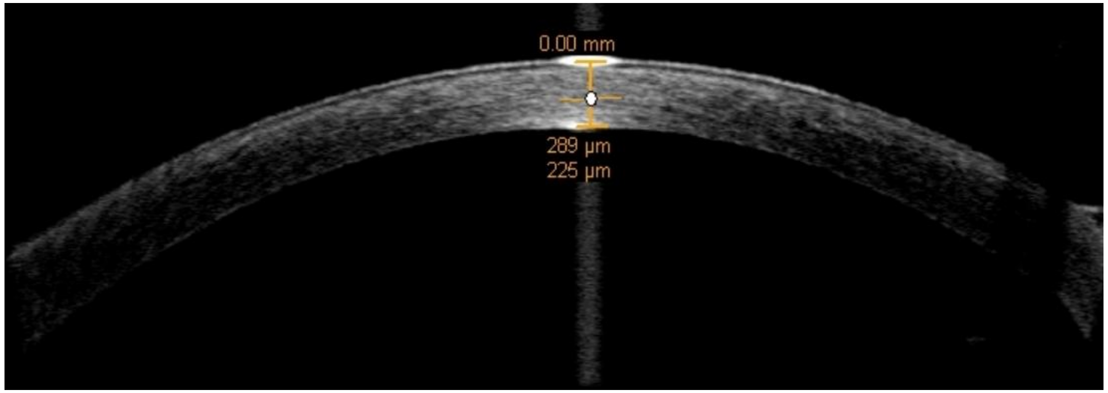
</td><td colspan="3">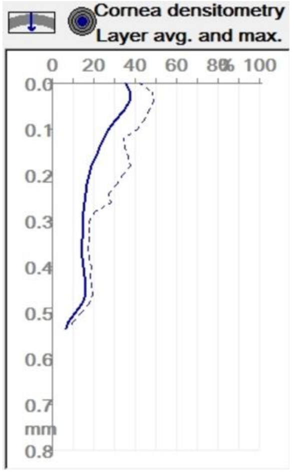
</td></tr>
<tr><td>289</td><td>514</td><td>56.23%</td><td>262</td><td>469</td><td>55.86%</td></tr>
<tr><td>44</td><td colspan="3">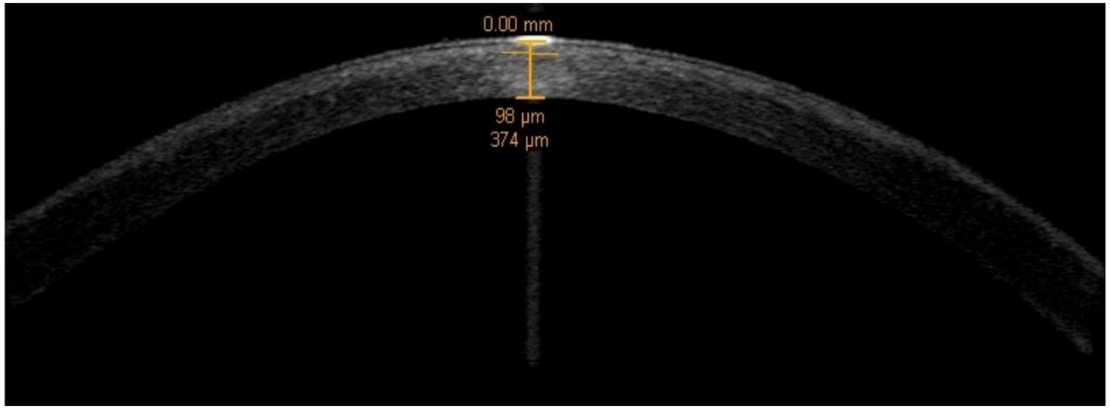
</td><td colspan="3">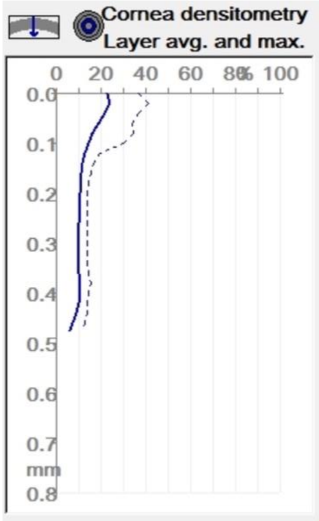
</td></tr>
</table>

	98	472	**20.76%**	80	475	**16.84%**
45	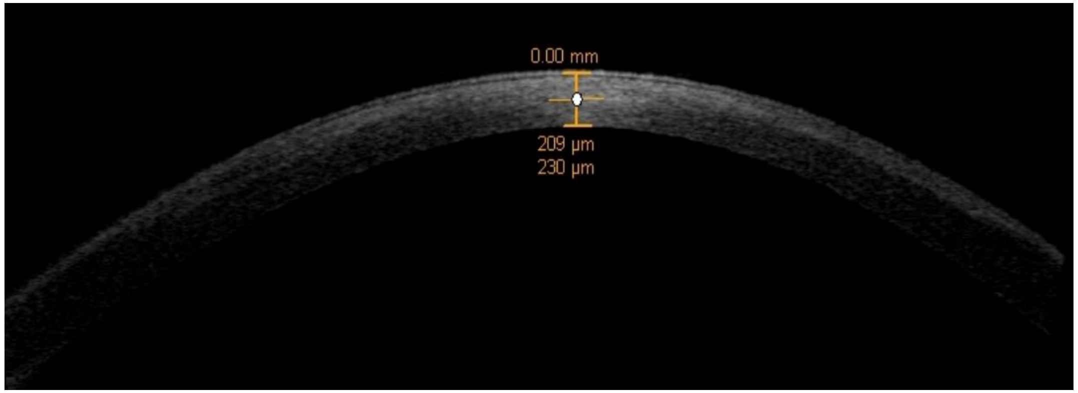			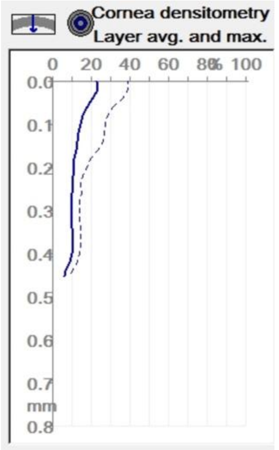		
	209	440	**47.50%**	177	453	**39.07%**
46	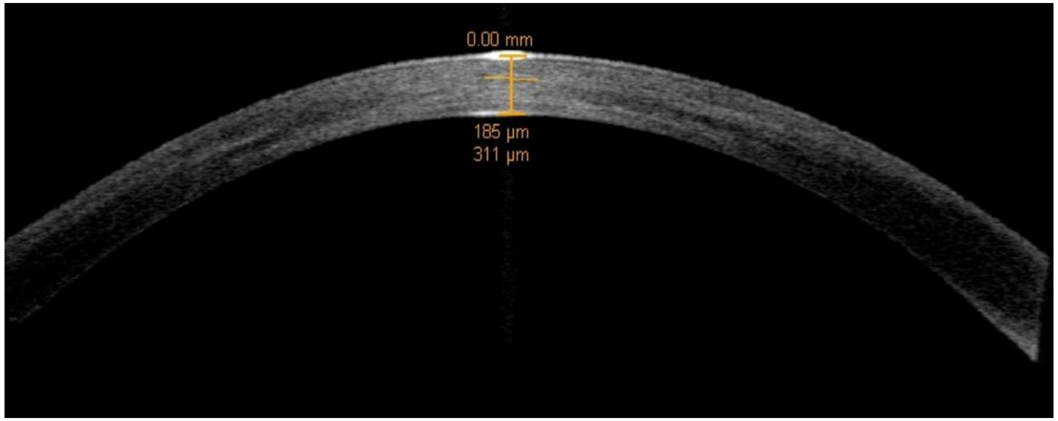			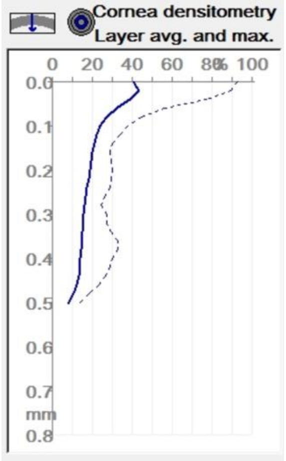		

	200	496	**40.32%**	200	503	**39.76%**
47	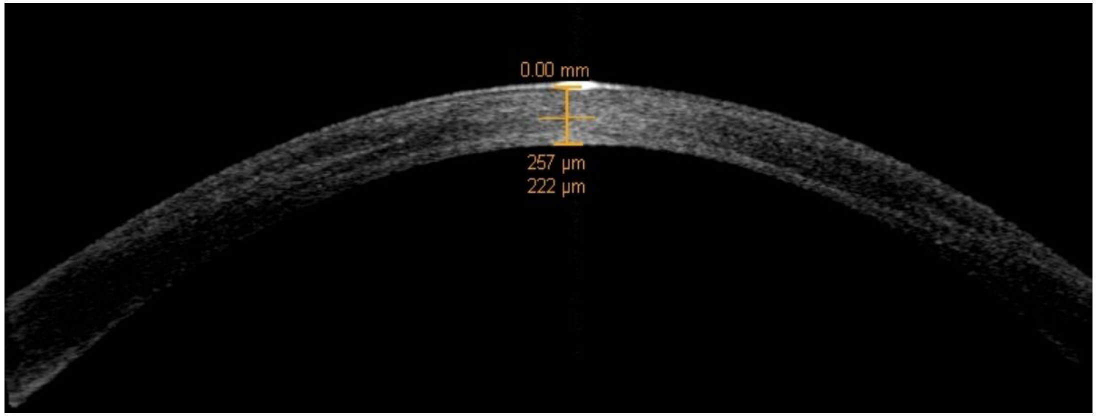			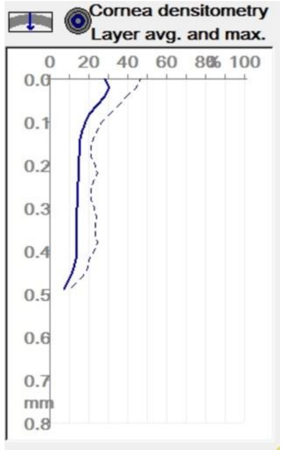		
	257	479	**53.65%**	221	491	**45.01%**
48	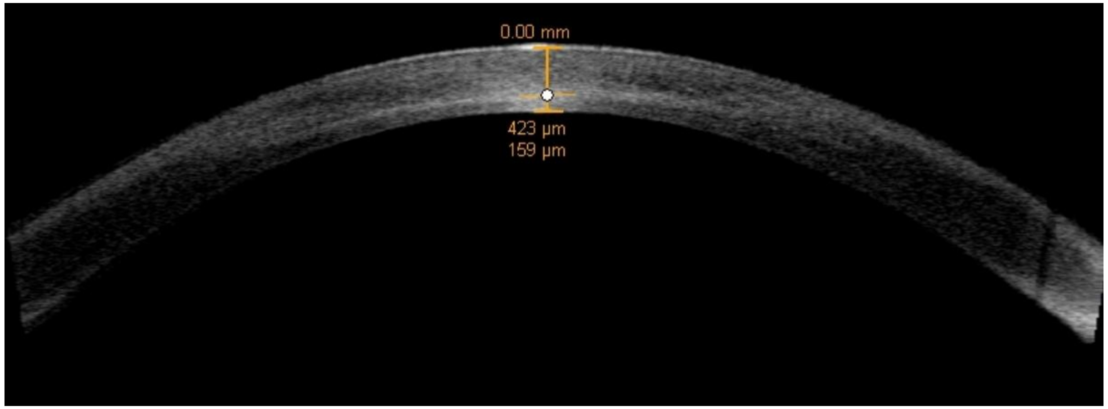			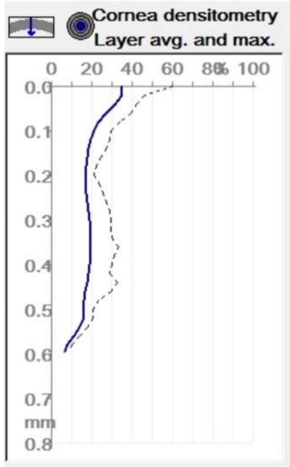		

<table>
<tr><td></td><td>436</td><td>585</td><td>74.53%</td><td>443</td><td>594</td><td>74.58%</td></tr>
<tr><td rowspan="2">49</td><td colspan="3">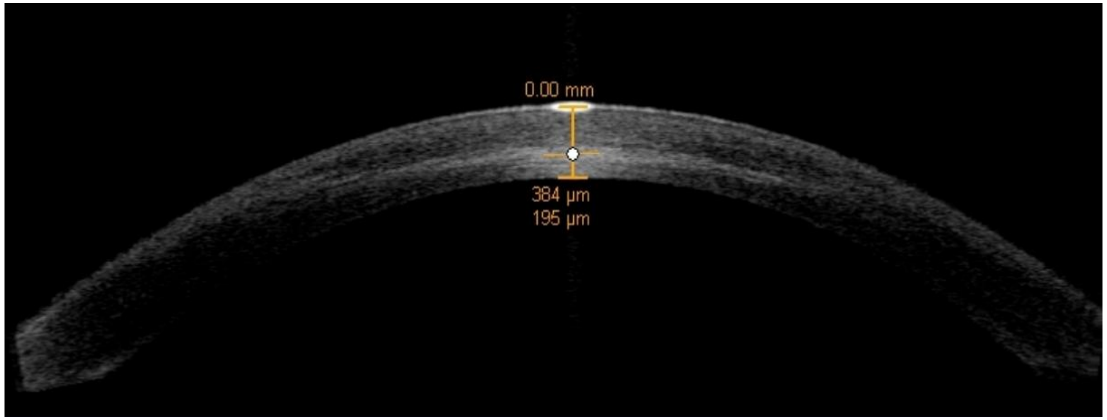
</td><td colspan="3">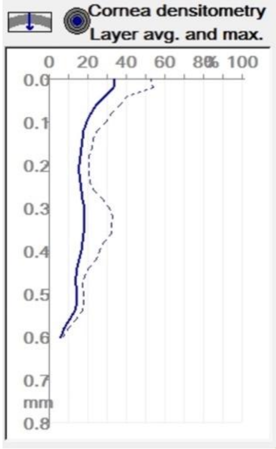
</td></tr>
<tr><td>384</td><td>575</td><td>66.78%</td><td>358</td><td>602</td><td>59.47%</td></tr>
<tr><td>50</td><td colspan="3">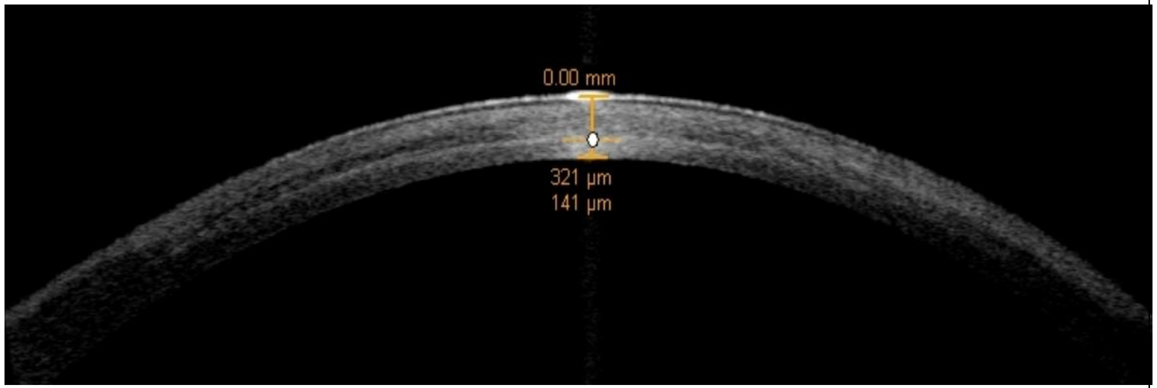
</td><td colspan="3">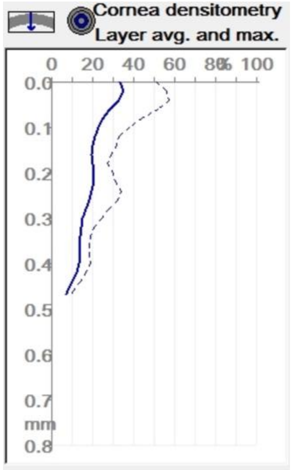
</td></tr>
</table>

	303	463	**65.44%**	259	467	**55.46%**
51	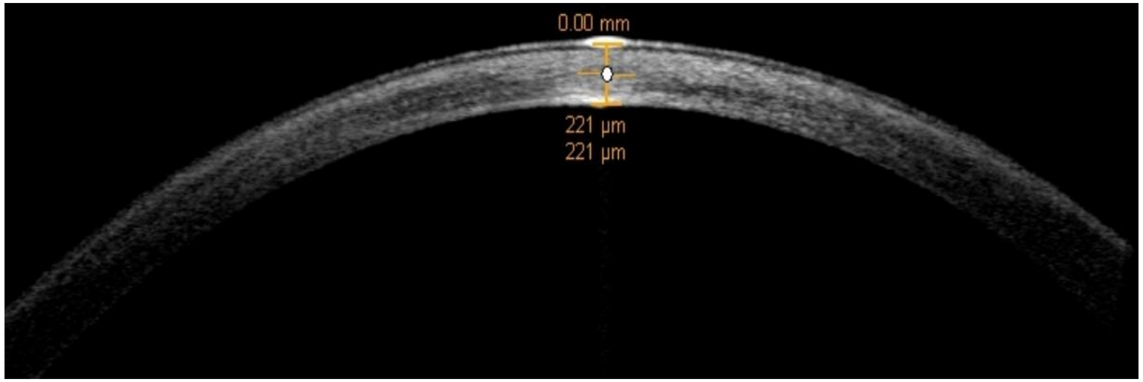			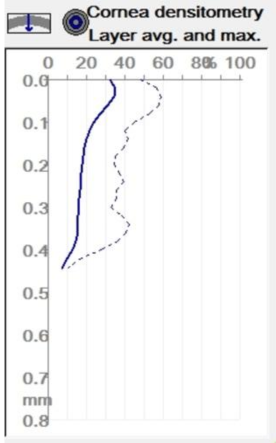		
	229	442	**51.81%**	240	444	**54.05%**
52	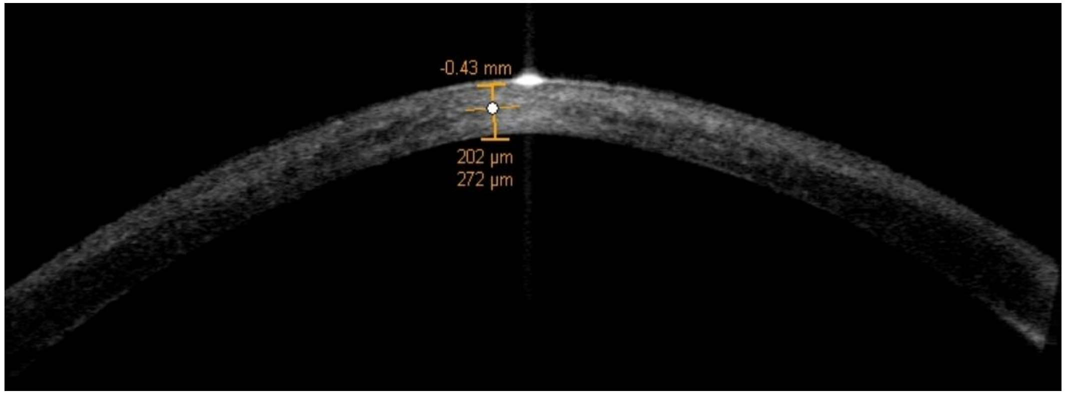			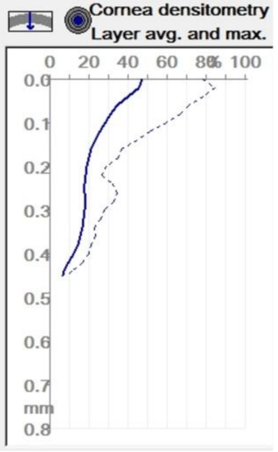		
	268	472	**56.78%**	262	451	**58.09%**

<table>
<tr>
<td rowspan="2">53</td>
<td colspan="3">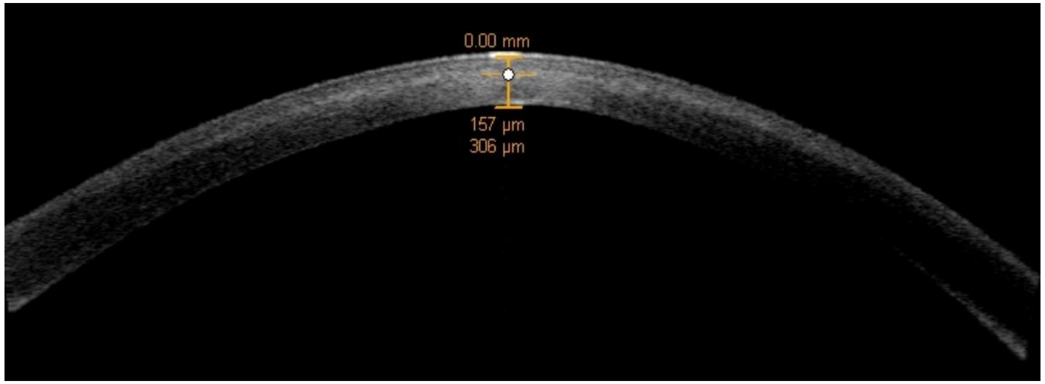
</td>
<td colspan="3">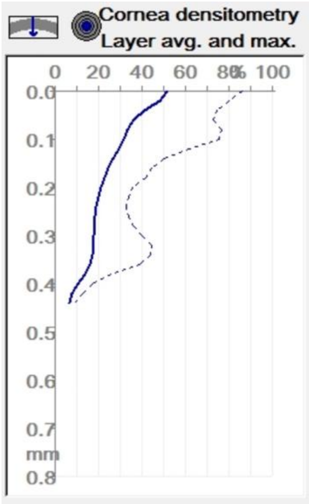
</td>
</tr>
<tr>
<td>157</td>
<td>463</td>
<td>33.91%</td>
<td>100</td>
<td>441</td>
<td>22.68%</td>
</tr>
</table>

Here is a portion of the raw data showing how we measure the DL by AS-OCT and the Max corneal densitometry graph (in 53 eyes);
Below each picture is the specific value of the DL;
Percentage (%) represents the ratio of DL depth to the corresponding corneal thickness.
